# Targeting FER Kinase Inhibits Melanoma Growth and Metastasis

**DOI:** 10.3390/cancers11030419

**Published:** 2019-03-24

**Authors:** Iordanka A. Ivanova, Shinthujah Arulanantham, Kevin Barr, Mario Cepeda, Katie M. Parkins, Amanda M. Hamilton, Danielle Johnston, Silvia Penuela, David A. Hess, John A. Ronald, Lina Dagnino

**Affiliations:** 1 Department of Physiology and Pharmacology, University of Western Ontario, London, ON N6A 5C1, Canada; iivanov3@uwo.ca (I.A.I.); sarulana@uwo.ca (S.A.); kevin.barr@schulich.uwo.ca (K.B.); dhess@robarts.ca (D.A.H.); 2 Children’s Health Research Institute, University of Western Ontario, London, ON N6C 2V5, Canada; 3 Lawson Health Research Institute, University of Western Ontario, London, ON N6C 2R5, Canada; 4 Department of Urology, Mayo Clinic, Rochester, MN 55902, USA; dr.mcepeda@gmail.com; 5 Department of Medical Biophysics, University of Western Ontario, London N6A 5C1, ON N6C 2R5, Canada; kparkin8@uwo.ca (K.M.P.); hamilton@robarts.ca (A.M.H.); jronald@robarts.ca (J.A.R.); 6 Robarts Research Institute, University of Western Ontario, London, ON N6A 5B7, Canada; 7 Department of Anatomy and Cell Biology, University of Western Ontario, London, ON N6A 5C1, Canada; Danielle.Johnston@schulich.uwo.ca (D.J.); Silvia.Penuela@schulich.uwo.ca (S.P.); 8 Department of Oncology, University of Western Ontario, London, ON N6A 5C1, Canada

**Keywords:** FER, tyrosine kinase, metastasis, tumor invasion

## Abstract

Melanoma is one of the most aggressive types of tumors and exhibits high metastatic potential. Fes-related (FER) kinase is a non-receptor tyrosine kinase that has been implicated in growth and metastasis of various epithelial tumors. In this study, we have examined the role that FER kinase plays in melanoma at the molecular level. FER-depleted melanoma cells exhibit impaired Wnt/β-catenin pathway activity, as well as multiple proteomic changes, which include decreased abundance of L1-cell adhesion molecule (L1-CAM). Consistent with the pro-metastatic functions of these pathways, we demonstrate that depletion of FER kinase decreases melanoma growth and formation of distant metastases in a xenograft model. These findings indicate that FER is an important positive regulator of melanoma metastasis and a potential target for innovative therapies.

## 1. Introduction

Cancer metastasis is a major contributor to cancer deaths, as there are very limited therapeutic modalities that can effectively prevent or target metastatic disease. Skin cancer is the most common malignancy in Canada and the United States, and its incidence is on the rise [1,2], with metastatic melanoma causing the majority of skin cancer-related deaths [3]. 

The identification of B-Raf kinase activating mutations in melanoma [4] enabled the development of small-molecule inhibitors specific for mutant B-Raf, and led to a breakthrough in metastatic melanoma treatment [5]. Other advances in metastatic melanoma treatment include the development of small-molecule inhibitors targeting MEK1/2 [6] and immunotherapies aimed at activating cytotoxic T cells [7,8]. Although the B-Raf inhibitors are very effective initially, leading to a pronounced decrease in the number and size of metastatic lesions, their effect is short-lived, and affected individuals quickly relapse because the tumors develop resistance to these drugs [9]. Thus, the need to better define the molecular basis of the metastatic behaviour in melanoma remains a critical step in the development of effective therapeutic interventions. 

The feline sarcoma (FES) and FES-related (FER) proteins constitute a distinct family of non-receptor tyrosine kinases involved in cell proliferation, cytoskeletal dynamics, and motility [10]. FER can be activated as a response to stimulation of various surface receptors, including those for epidermal growth factor and platelet-derived growth factor [11,12]. Upon activation by growth factors, FER can associate with and phosphorylate the adherens junction molecule p120-catenin and the actin binding protein cortactin [11,12]. Phosphorylation of cortactin by FER regulates migration in various cell types [13,14,15]. 

Several lines of evidence support a role of FER in malignant progression. FER promotes cell cycle progression in prostate and breast carcinoma, as well as in acute myeloid leukemia cells [16,17]. In prostate carcinoma cells, FER is highly expressed relative to normal prostate epithelium, and regulates proliferation by activating signal transducer and activator of transcription 3 [18]. Increased FER expression has also been linked to invasion and dissemination of metastatic hepatocellular carcinoma cells [19]. FER also plays key roles in breast, lung and ovarian carcinoma metastasis, and is an independent predictor of survival in individuals suffering from invasive breast cancer [20,21,22]. Thus, FER is likely a strong pro-metastatic factor in a variety of epithelial human tumors. 

Here, we have addressed the possibility that FER may be a broad regulator of tumorigenesis and have investigated its regulation of melanoma cell behavior. We now demonstrate that FER promotes L1-CAM expression and metastasis in melanoma cells, placing FER as a potential target in this tumor type.

## 2. Results

### 2.1. Analysis of FER Kinase in Human Melanoma Tumors 

To begin to investigate whether FER kinase may be associated with melanoma growth and/or progression, we first examined the relevant clinical datasets in the Cancer Genome Atlas (TCGA). This analysis revealed a virtual absence of FER gene mutations in this tumor type (Figure S1A). Our analysis also showed consistent FER expression and transcript abundance in all samples reported, irrespective of the tumor grade, with higher mRNA levels detected in a small subset of these tumors (Figure S1B,C). We also measured FER protein abundance in a small sample of metastatic melanoma lesions, and observed variable, but readily detectable FER levels (Figure S1D). We conclude that FER is consistently present in melanoma tumors, although at somewhat variable levels.

### 2.2. S-Phase Abnormalities in FER-Deficient Melanoma Cells 

**Figure 1:**
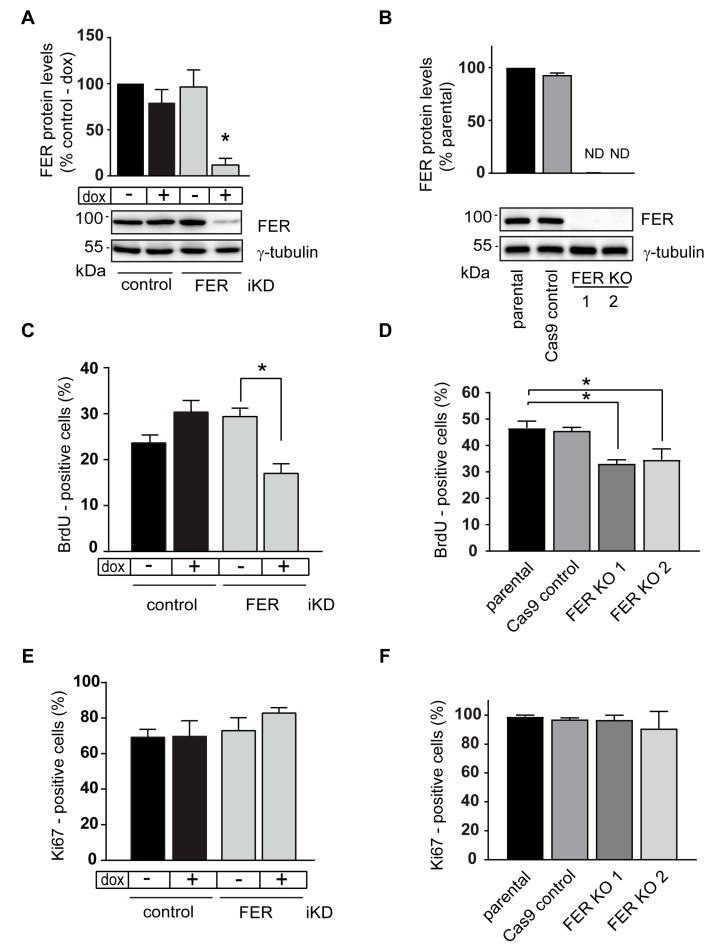
S-phase abnormalities in FER kinase-deficient melanoma cells. (A) Inducible knockdown (iKD) of FER in melanoma cells. 131/4-5B1 cells were transduced with lentivirus encoding doxycycline (dox)-inducible control or FER shRNA. Cells were cultured for 120 h in the absence (−) or presence (+) of 2 µg/mL doxycycline (dox) and FER kinase was detected by immunoblot. γ-tubulin was used as a loading control. (**B**) Generation of FER knockout (KO) melanoma cells. A375-MA2 melanoma cells were transfected with plasmids encoding wild type Cas9 endonuclease and single-guide RNAs targeting FER exon 1 or exon 3. Single cell clones were isolated and successful targeting of FER was confirmed by immunoblot. Histograms represent mean FER protein levels ± SEM (*N* = 3) normalized to γ-tubulin and expressed relative to control—dox cells (**A**) or to parental cells (**B**), which are set to 1. (**C**) Effect of FER kinase silencing on S-phase. 131/4-5B1 control and FER iKD cells were cultured in medium with or without 2 μg/mL of dox for 120 h or (**D**) A375-MA2 parental, Cas9 control and FER KO cells were cultured for 48 h. Then, cells were incubated in medium containing 10 μM of BrdU for 2 h. The cells were processed for immunofluorescence microscopy using an anti-BrdU antibody. (**E**) Effect of FER kinase silencing on Ki67 expression. 131/4-5B1 control and FER iKD cells were cultured in medium with or without 2 μg/mL of dox for 120 h. (**F**) A375-MA2 parental, Cas9 control and FER KO cells were cultured for 48 h. Then, cells were processed for immunofluorescence microscopy, using an anti-Ki67 antibody. The histograms represent the fraction of Ki67- or BrdU-positive cells in each treatment group, expressed as the mean ± SEM (*N* = 3). * represents *p* < 0.05 (One-way ANOVA, Tukey’s post-hoc test).

The 131/4-5B1 [23] and A375-MA2 [24] melanoma lines were originally developed by selecting the population of melanoma cells that can give rise to distant metastases in the lungs and brain following transplantation in immunodeficient mice. To investigate the role that FER kinase plays in melanoma growth, we generated FER-deficient melanoma lines using two different approaches. In the first approach, we stably modified 131/4-5B1 cells using either dox-inducible FER-targeting or control, non-targeting shRNA-encoding lentiviruses. Culture of FER-inducible knockdown (iKD) 131/4-5B1 cells in the presence of dox for 120 h decreased FER protein levels by about 90%, relative to FER levels in either untreated FER iKD cells, or in control cells expressing non-targeting shRNAs, irrespective of the presence or absence of dox (Figure 1A). Thus, in this system, a marked dox-dependent reduction in FER protein levels is observed specifically in cells transduced with FER-targeting shRNA sequences. 

As a second approach, we used CRISPR/Cas9 gene editing. We generated two different monoclonal A375-MA2 FER knockout (KO) cell lines, by targeting either exon 1 or exon 3 in the *FER* gene. We also generated the corresponding control lines by transiently transfecting the parental A375-MA2 line with the Cas9-encoding plasmid, but without FER-targeting sgRNAs. Following this same approach, we were unable to generate CRISPR/Cas9 FER-edited 131/4-5B1 lines. Analysis of the clonal A375-MA2 lines selected revealed readily detectable FER protein in the parental and control A375-MA2 cells, whereas FER was undetectable in all the KO lines (Figure 1B). 

We next examined the consequences of FER deficiency on the proliferative capacity of the melanoma lines we generated. Labeling of these cells with BrdU revealed a 25–40% decrease in the fraction of cells in S-phase (Figure 1C,D), indicating that absence of FER results in perturbations in the cell cycle. Of note, all cell populations exhibited similar proportions of Ki67-positive cells (70–80%, Figure 1E,F). Collectively, our data indicate that FER modulates processes involved in normal transit through S-phase, although it is not essential to maintain melanoma cells in an active proliferation state.

### 2.3. FER Regulates Melanoma Cell Motility 

**Figure 2:**
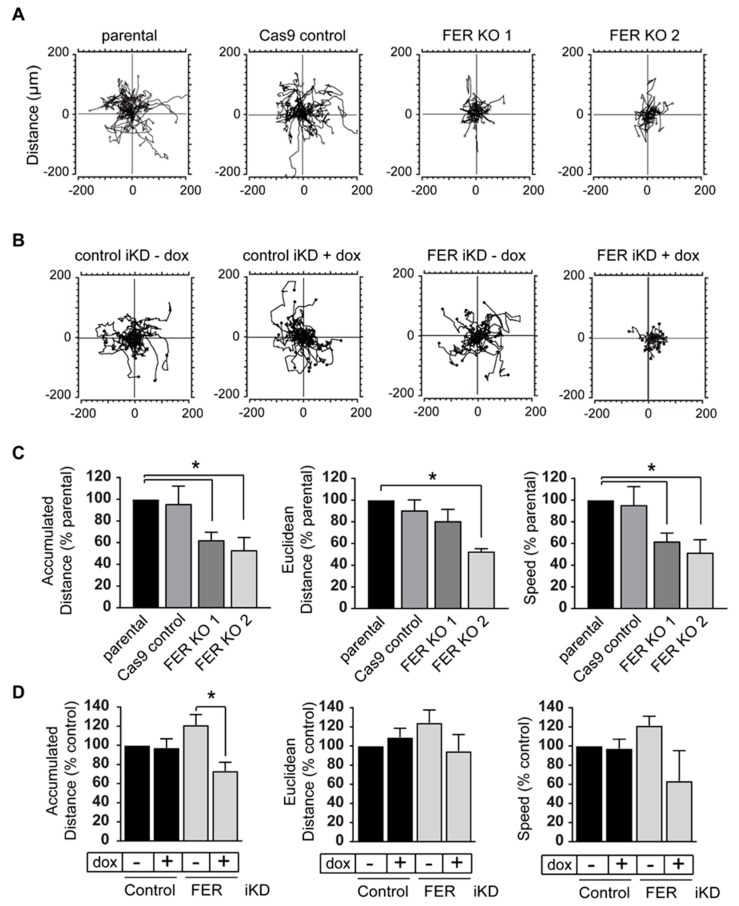
FER regulates melanoma cell motility. (**A**) A375-MA2 parental, Cas9 control and FER KO cells were cultured in medium containing 0.5% FBS for 96 h or (**B**) Control and FER iKD 131/4-5B1 cells were cultured in medium with or without 2 μg/mL of dox for 120 h. Then, cells were cultured on μ-Dishes coated with laminin 332. Cells were imaged for 16 h using time-lapse videomicroscopy and analyzed using Image J (NIH) and the Chemotaxis and Migration Tool Software (ibidi). (**A**,**B**) Representative migratory paths of FER-expressing and FER-deficient cells on laminin 332. The initial migratory point of a cell is represented as (0,0) and the final point is illustrated as a closed circle. (**C**,**D**) At least 100 cells were tracked per cell line or condition per experiment. Histograms represent mean ± SEM (*N* = 3) accumulated (total) distance, Euclidean distance (straight linear distance from the initial to final migratory point) and speed of cells normalized to those of parental cells (**C**) or control – dox cells (**D**). * represents *p* < 0.05 (One-way ANOVA, Tukey’s post hoc test).

The propensity of melanoma cells to metastasize has been attributed, in part, to their ability to interact with and modify their surrounding extracellular matrix, and to their imprinted high migratory capacity, arising from the embryonic neural crest cells that give rise to melanocytic cells [25]. Cultured melanocytes exhibit marked differences in migratory capacity, depending on the substrate on which they are seeded [26]. Consequently, we first determined the effect of various extracellular substrates on motility of parental 131/4-5B1 cells using time-lapse videomicroscopy. We observed limited motility in cells cultured either without any added exogenous matrix or on collagen I. Under these conditions, the cells were able to migrate a total distance of about 180 μm in a 16-h period, with an average speed of 0.19 μm/min (Figure S2A). In contrast, cells cultured on laminin 332 matrix, which is one of the principal components of the basement membrane that separates the dermis from the epidermis, displayed significant increases in cell motility, with a mean speed of ~0.3 μm/min (Figure S2A). Consequently, all additional cell motility experiments were conducted with cells seeded on laminin 332 matrix. Under these conditions, FER-deficient cells exhibited significant decreases in total distance migrated as evidenced by the shorter migratory paths of FER KO and FER iKD cells, relative to controls (Figure 2A,B). Specifically, we found that accumulated migration distance was reduced by approximately 40% in the FER KO cells, which is likely a consequence of the observed 40–50% reduction in migration speed (Figure 2C and Figure S2B). Similar results were observed in FER iKD melanoma cells, indicating that substantially reducing FER protein levels is sufficient to impair melanoma cell motility (Figure 2D and Figure S2C). In contrast, loss or reduction of FER protein levels had little, if any, effect on Euclidean distance (the linear measure of the distance between the initial and final cell position) migrated by the melanoma cells, (Figure 2C,D and Figure S2B,C), indicating that loss of FER does not significantly affect the directionality of melanoma cell movement under the conditions of our experiments. 

Cell interactions with the surrounding extracellular matrix modulate not only motility, but also adhesion. Thus, we next assessed the consequences of FER depletion on melanoma cell attachment in the presence and absence of laminin 332 matrix. To this end, we seeded FER-expressing and FER-depleted cells and measured their ability to adhere at timed intervals thereafter. The adhesion of FER iKD and FER KO cells was undistinguishable from that of their respective controls, irrespective of the presence or absence of exogenously supplied laminin 332 (Figure S3). Collectively, our results show that FER positively modulates melanoma cell proliferation and motility, two processes that are essential for tumor progression and metastasis. 

### 2.4. Discovery of Altered Cellular Signaling Networks in FER-Deficient Cells

To gain insight into the pathways modulated by FER in melanoma cells, we conducted a proteomic screening using reverse phase protein arrays (RPPA). We compared changes in protein expression and activation between control and FER iKD 131/4-5B1 cells cultured in the presence or absence of dox. Because tetracyclines can affect mitochondrial translation [27], we first identified dox-associated changes, irrespective of FER status (Figure S4A). These changes included upregulation of eukaryotic translation initiation factor 4E-binding protein 1 and programmed cell death 4, and downregulation of phospho-ribosomal protein S6. Dox treatment also reduced levels of cyclin B1, cdk 1, phospho-Akt and phospho-Rb. Proteins involved in cell metabolism, including glycogen synthase and monocarboxylate transporter 4 were up- or downregulated, respectively, in cells treated with dox. We also found that dox downregulated stress response proteins, such as poly-ADP ribose polymer and superoxide dismutase 2. In this manner, we were able to identify and exclude dox-associated changes from further analysis. 

**Figure 3:**
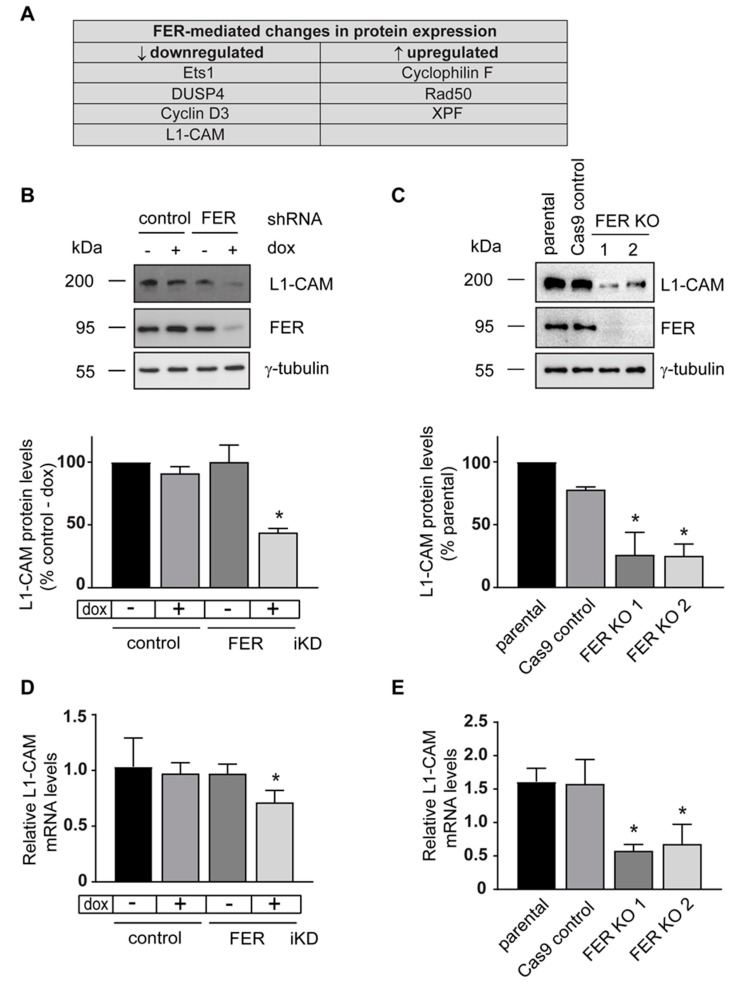
Functional proteomics reveal that L1-CAM is regulated by FER kinase in melanoma cells. (**A**) Control and FER iKD 131/4-5B1 cells were cultured in medium with or without 2 μg/mL of dox for 120 h. The levels of 306 proteins in control- and FER shRNA-expressing melanoma cell lysates were compared using reverse phase protein array (RPPA) analysis. The table shows proteins that were up- or downregulated in FER-depleted cells relative to control cells. L1-CAM protein levels decrease in FER-depleted melanoma cells. L1-CAM protein levels were analyzed by immunoblot in (**B**) 131/4-5B1 control and FER iKD cells and (**C**) A375-MA2 parental, Cas9 control and FER KO cells. Histograms represent mean L1-CAM protein levels ± SEM (*N* = 3) normalized to γ-tubulin and expressed relative to control—dox cells (**B**) or to parental cells (**C**). FER-depletion leads to reduced L1-CAM mRNA levels. L1-CAM mRNA levels were analyzed by real-time quantitative PCR in (**D**) 131/4-5B1 control and FER iKD cells and (**E**) A375-MA2 parental, Cas9 control and FER KO cells. Histograms represent mean L1-CAM mRNA levels ± SEM (*N* = 3) normalized to HMBS or HPRT1. * represents *p* < 0.05 (One-way ANOVA, Tukey’s post hoc test).

Profiling of cell lysates also revealed altered levels of 30 proteins specifically associated with FER depletion, which included regulators of cell proliferation, apoptosis, DNA repair and MAPK signaling (Figure S2B), indicating that depletion of FER results in molecular changes across multiple pathways. Consistent with the observed role of FER in promoting cell proliferation, we found decreased cyclin D3 levels in FER-depleted cells (Figure 3A and Figure S2B). Protein levels of c-Kit, c-Met, and HER3 receptors, and of the Syk tyrosine kinase were increased upon FER downregulation, although no changes in phosphorylated c-Met were detected (Figure S4B). FER silencing also affected proteins involved in DNA repair, including excision repair cross-complementation group 1, *xeroderma pigmentosum* group F-complementing protein and Rad50, which were upregulated, as well as phosphorylated *ataxia telangiectasia*-mutated and Rad3-related kinase, which was downregulated. Although we did not observe detectable changes in apoptosis in FER-deficient cells cultured under optimal conditions, the apoptosis-associated proteins porin and cyclophilin F were upregulated. Significantly, we also found that L1-CAM was downregulated in FER-depleted cells. L1-CAM has key roles in cell-cell and cell-matrix interactions [28], and L1-CAM expression is positively correlated with melanoma progression and metastatic potential [29,30,31]. We confirmed by immunoblot analysis that L1-CAM protein levels are substantially decreased in FER-depleted 131/4-5B1 iKD and A375-MA2 FER KO cells by 56% and 75%, respectively (Figure 3B,C). As L1-CAM can be regulated both transcriptionally and post-translationally, we tested *L1CAM* mRNA expression in FER-deficient cells and noted a significant decrease in *L1CAM* mRNA abundance by 25% and 64%, respectively, in FER iKD and FER KO cells (Figure 3D,F). We also assessed if L1-CAM turnover was altered in FER-deficient cells. For these studies, we cultured control and FER iKD 131/4-5B1 cells in the presence cycloheximide and dox. We found that the apparent half-life of L1-CAM in the presence of dox was decreased by about 25% (Figure S5). Together, these observations are consistent with the notion that FER modulates L1-CAM by multiple mechanisms.

### 2.5. FER Requirement for β-Catenin/T Cell Factor-Lymphoid Enhancer Factor (TCF-LEF) Transcriptional Activity 

**Figure 4:**
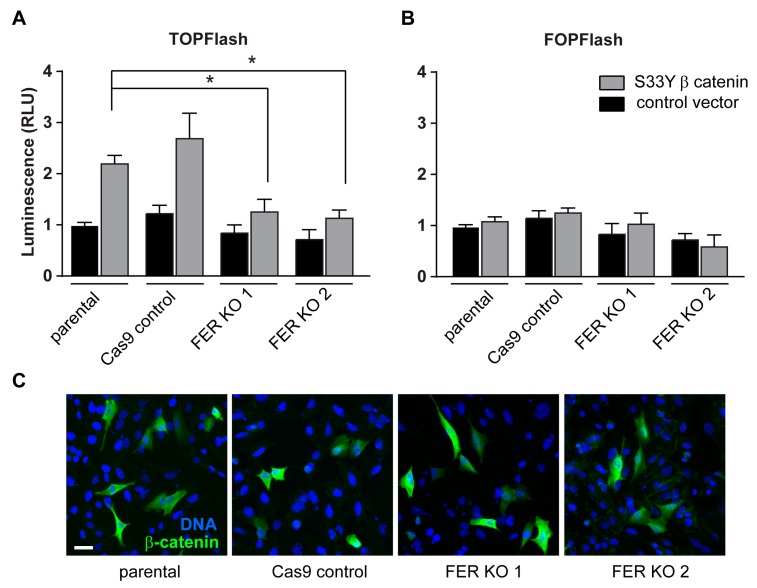
Reduced TCF/LEF activity in FER deficient melanoma cells. A375-MA2 parental, Cas9 control and FER KO cells were transfected with (**A**) TOPFlash or (**B**) FOPFlash firefly luciferase reporter plasmids containing optimal or mutant TCF/LEF binding sites, respectively. Cells were co-transfected with either a control vector or a vector expressing a stable β-catenin S33Y mutant and *Renilla* luciferase to normalize for transfection efficiency. Histograms represent mean ± SEM (*N* = 3) relative luciferase activity and were normalized to that in parental cells * represents *p* < 0.05 (One-way ANOVA, Tukey’s post hoc test). (**C**) A375-MA2 parental, Cas9 control and FER KO cells were transfected with a vector expressing a stable β-catenin S33Y mutant and processed for immunofluorescence microscopy using an anti-β-catenin antibody. DNA was visualized with Hoescht 33342. Bar = 10 µm.

The transcription of several genes encoding factors implicated in melanoma growth, including *L1CAM*, can be modulated by β-catenin/TCF-LEF. Therefore, we next examined the effect of FER depletion on Wnt/β-catenin-dependent transcriptional activation of TCF-LEF. Control and FER KO melanoma cells were transfected with TOPFlash or FOPFlash firefly luciferase reporter vectors containing, respectively, eight functional or mutated TCF/LEF binding sites. Baseline TCF/LEF reporter activity was similarly low in unstimulated control and in FER-depleted cells (Figure 4A).

To examine if FER modulates Wnt/β-catenin cellular responses, we next investigated the effect of a β-catenin S33Y mutant on TCF/LEF activity. This phosphorylation-deficient mutant is not a substrate for glycogen synthase kinase-3β and, consequently, exhibits decreased degradation, promoting stimulation of TCF/LEF-mediated transcription. β-catenin S33Y was expressed at similar levels in control and in FER KO cells (Figure S6) and was readily detected both in regions adjacent to the plasma membrane and in the nucleus of both cell types (Figure 4B,C). Notably, although β-catenin S33Y increased TCF/LEF reporter activity 2.5-fold in control cells, it was without effect in FER KO cells (Figure 4A). Thus, FER positively modulates β-catenin/TCF-LEF-dependent transcription in melanoma cells, through mechanisms independent of β-catenin degradation or nuclear import.

### 2.6. Role of FER in Melanoma Cell Invasion

Melanoma is thought to metastasize through a series of processes that involve phenotype switching, first characterized by the ability to invade surrounding tissues. Invasion is then followed by intravasation, survival in the blood stream, extravasation and growth at secondary sites [32]. Therefore, we first assessed the ability of FER-deficient cells to invade surrounding tissues using melanoma cells xenografted onto the chorioallantoic membrane (CAM) of ex ovo chicken embryos. 

**Figure 5:**
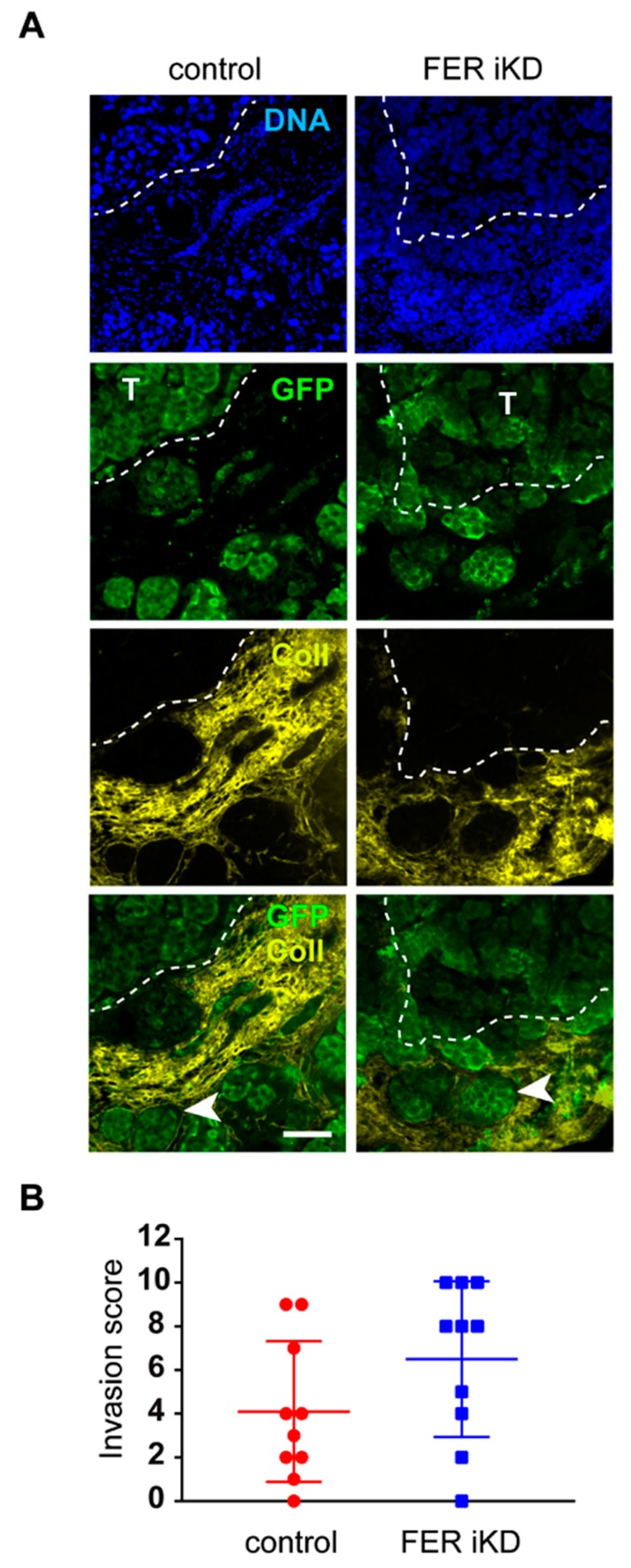
Effect of FER downregulation on melanoma cell invasion. 131/4-5B1 control and FER iKD cells were treated with 2 µg/mL doxycycline for 120 h. Then, cells were suspended in Cultrex and grafted on the chorioallantoic membranes (CAMs) of E11 chicken embryos. On E18, tumors were excised and processed for further analysis. (**A**) Representative sections of control and FER iKD tumors. GFP and collagen III (Coll) antibodies were used to detect melanoma cells and delineate the CAM, respectively. The dotted line outlines the tumor-CAM interface. The arrow indicates tumors cells that have invaded the CAM mesoderm. T: tumor, bar = 100 μm. (**B**) Ten serial sections from control and FER iKD tumors (*n* = 10) were examined. Sections with GFP immunoreactivity within collagen III-positive areas were scored as showing melanoma cell invasion. The dot plot illustrates the invasion scores (number of tumor tissue sections, out of 10, showing invasion).

This model was chosen because it supports interactions between melanoma cells and surrounding stroma in the presence of robust vascularization, features that are difficult to mimic in cell culture systems. For these experiments, 131/4-5B1 control and FER iKD cells that had been pre-treated with dox for 5 days were suspended in basement membrane extract (Cultrex) and grafted ex ovo onto the CAM of chick embryos. We observed that FER-expressing 131/4-5B1 cells produced tumors on the chorionic epithelium of the CAM that were readily visible on histological examination 7 days post-grafting (Figure 5A and Figure S7A,B). Further, green fluorescent protein (GFP)-positive 131/4-5B1 were readily distinguished from CAM stroma, which was visualized using an antibody that specifically recognizes chicken collagen type III (Figure 5A and Figure S7C). The identity of cells with positive GFP immunoreactivity was further confirmed by the presence of the melanoma marker PMEL (Figure S7D). We were also able to detect FER-deficient cells in the CAM stroma (Figure 5A). To assess whether we could detect any differences in the invasion ability of these cells, we analyzed 10 evenly distributed sections for each tumor, assessing the presence of GFP-positive cells in the collagen-positive CAM mesoderm, as previously described by Lokman et al. [33]. We then assigned an invasion score to each tumor based on the number of sections in which GFP-positive cells were detected in the CAM mesoderm. This analysis revealed no significant differences in invasion scores between FER-expressing and FER-deficient melanoma cells (Figure 5B). Although this approach did not allow us to quantify subtle differences between tumor types, our results are consistent with the notion that normal FER protein levels are not required for melanoma invasion.

### 2.7. Reduced Tumor Growth and Formation of Distant Metastases in FER-Deficient Melanoma

To evaluate alterations in tumor growth and formation of metastatic lesions resulting from FER downregulation, we used a mouse xenograft model. To this end, we generated control and FER iKD 131/4-5B1 cells that also stably express firefly luciferase (Figure S8). To test the effects of FER silencing on tumor growth and metastasis in vivo, we subcutaneously injected control and FER iKD cells that had been cultured in the absence of dox into NSG immunodeficient mice. This approach allowed us to avoid any potential differences between FER-expressing and FER-deficient cells due to alterations in engrafting capacity. We began by determining the ability of the luciferase-modified 131/4-5B1 control and FER iKD cells to form primary tumors. Palpable tumors were first detected five weeks following implantation with both cell types (Figure S9A). At this time, we initiated a dox-containing diet for five additional weeks and observed that tumor-associated luminescence was lower in mice inoculated with 131/4-5B1 control cells relative to those that received FER iKD cells. Under these conditions, we detected metastasis signals only in mice bearing 131/4-5B1 control cells (Figure S9A). 

To further evaluate FER-associated differences in melanoma metastatic potential, we next inoculated mice with luciferase-expressing control and FER iKD 131/4-5B1 cells, and one day post-injection, the mice began to receive dox-containing diet to induce shRNA expression. Because peak dox plasma concentrations in mice are attained as early as 2 days following dox administration in feed [34], this strategy allowed us to impair FER expression shortly after melanoma cell engrafting.

**Figure 6:**
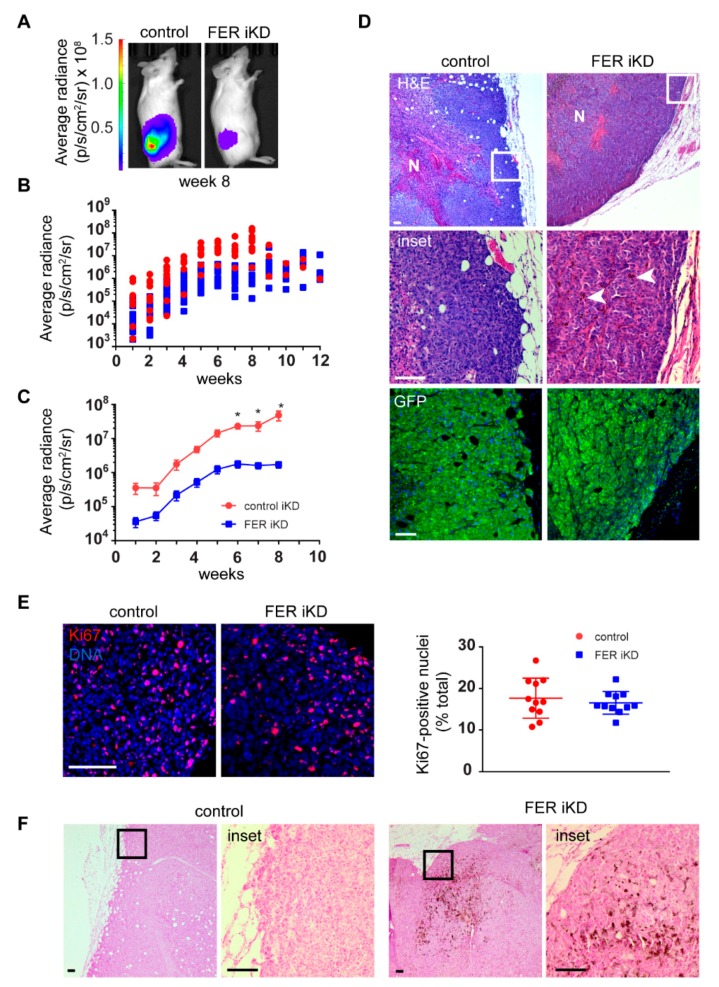
FER regulates melanoma primary tumor growth. (**A**) Bioluminescence imaging (BLI) of melanoma cells in vivo. Luciferase-expressing control and FER iKD melanoma cells were orthotopically transplanted in NSG immunodeficient mice, which were fed a dox-containing diet to induce shRNA expression. Melanoma cells were detected in live animals using BLI. (**B**) FER downregulation reduces growth in primary melanoma tumors. Following melanoma cell implantation, mice were imaged weekly and the signal detected from primary tumors in individual mice was quantified. (**C**) Mean radiance ± SEM (*n* = 11) of control and FER iKD tumors 8 weeks post-transplantation. * represents *p* < 0.05 (Two-way repeated measures ANOVA, Sidak’s post hoc test). (**D**) Histological analysis of control and FER-deficient primary melanoma tumors. Tumor tissue sections were stained with hematoxilyn and eosin (H&E) and visualized with light microscopy. Necrotic areas (N) were present in both control and FER-deficient tumors. Tumor cells at the periphery were viable (insets) and pigmented tumor cell clusters (arrows) were detected in FER-deficient tumors. Immunofluorescence staining using an anti-GFP antibody was performed on adjacent tumor sections to confirm tumor cell viability (shRNA-expressing melanoma cells also express GFP). (**E**) Control and FER iKD tumors were processed for immunofluorescence microscopy with an anti-Ki67 antibody. DNA was visualized with Hoescht 33342. The percentage of Ki67-positive nuclei was determined (*n* = 11). (**F**) Sections from control and FER iKD tumors were processed with Fontana-Masson staining and visualized with light microscopy to highlight melanin-producing melanoma cells. Bar = 100 µm.

Both control and FER iKD subcutaneous tumors were detected by bioluminescence imaging (BLI) as early as one-week post-injection (Figure 6B). FER-expressing tumors grew rapidly over an 8–10-week period, as indicated by the exponential increase in their luminescence. In stark contrast, FER iKD tumor growth was substantially lower within the 8–10-week interval (Figure 6A–C). For these studies, the experimental endpoint was established as soon as detectable metastases were present or when primary tumor volumes reached 1 cm^3^. For this reason, the experimental endpoints for mice bearing FER-expressing and FER-deficient tumors were, respectively, 8–10 and 11–13 weeks. Because of the additional growth period allowed for FER iKD primary tumors, their endpoint weights were higher than those in control tumors (Figure S9B). Quantification of FER protein abundance in primary tumors at endpoint revealed significantly lower levels in FER iKD specimens, in spite of potential contributions of host-derived cells likely present in the xenografts (Figure S9C). Histological analysis at endpoint revealed that both control and FER iKD primary tumors contained densely packed, GFP-positive viable melanoma cells at their periphery (Figure 6D). Both tumor types were poorly vascularized and exhibited large necrotic areas at their cores. In agreement with our observations using CAM invasion assays, areas of local invasion into surrounding host tissue were also present for both tumor types. At endpoint, control and FER iKD primary tumors contained similar proportions of Ki67-positive cells (approximately 20%; Figure 6E), consistent with their behaviour in culture. Notably, we observed clusters of melanin pigmented cells in FER iKD, but not in control, tumors (Figure 6D,F). Thus, FER depletion decreases melanoma tumor growth over time, and is associated with activation of melanin synthesis-associated pathways in subsets of FER-deficient cells in vivo.

**Figure 7:**
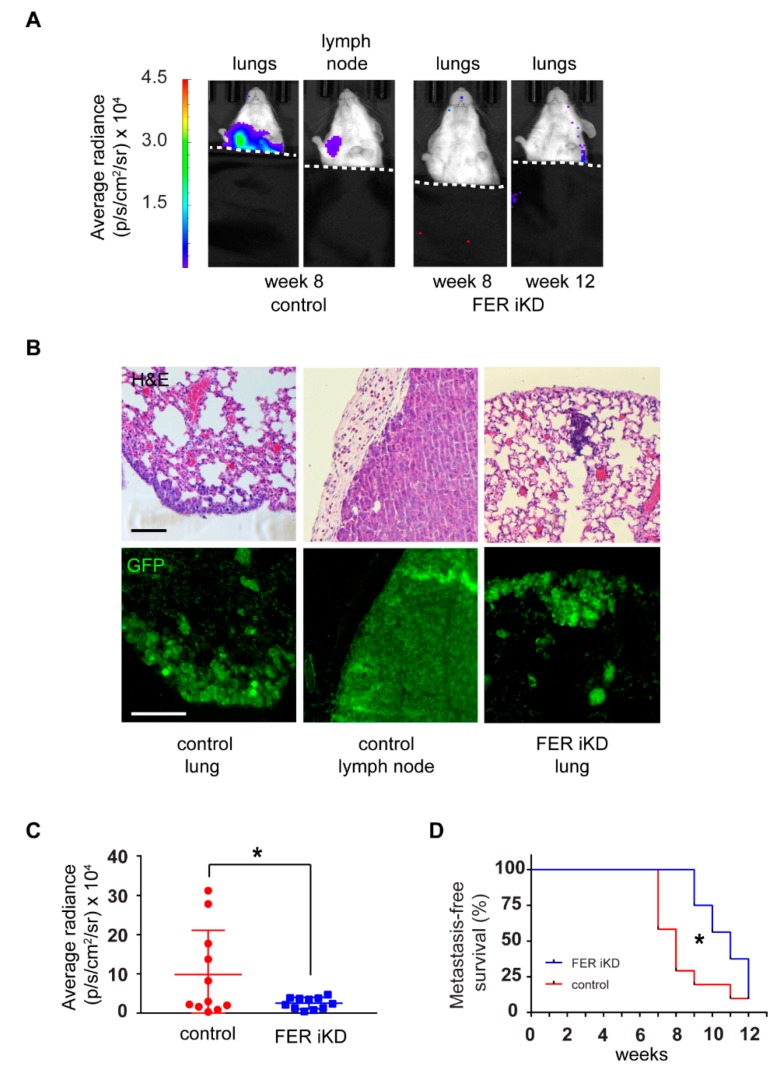
FER downregulation inhibits formation of melanoma distant metastases. (**A**) Bioluminescence imaging (BLI) of melanoma cell distant metastases in control and FER iKD tumor-bearing mice. The mice were covered during imaging to prevent interference from the signal emitted from cells in the primary tumor (the dotted line delineates the exposed and covered areas). (**B**) Lung and lymph node tissue sections were stained with hematoxilyn and eosin (H&E) and visualized with light microscopy. Immunofluorescence staining using an anti-GFP antibody was performed on adjacent tissue sections to confirm distant metastases of melanoma cells. Bar = 100 µm. (**C**) Mean radiance ± SEM (*n* = 11) of distant metastases of control and FER iKD tumor-bearing mice at endpoint. * represents *p* < 0.05 (Welch’s t test). (**D**) Metastasis-free survival analysis of control and FER iKD tumor-bearing mice (*n* = 11). * represents *p* < 0.05 (Mantel-Cox test).

We also assessed the consequences of FER downregulation on formation of metastatic foci, using BLI to monitor tumor-bearing mice longitudinally. We found that 40% of mice with FER-expressing tumors developed BLI-detectable metastases in the lungs and lymph nodes approximately 7 weeks following tumor cell injection, and by 11 weeks all animals in this group exhibited evidence of metastatic spread (Figure 7D). In contrast, we only began to detect metastases in mice bearing FER iKD tumors after 9 weeks (Figure 7A,B). The metastatic burden at endpoint (8–10 weeks for control tumors or 11–13 weeks for FER iKD tumors) was greater in mice with FER-expressing tumors, as indicated by the significantly higher luminescence signals detected in control vs FER iKD metastases (Figure 7C), despite the larger size that FER iKD primary tumors had reached at endpoint (Figure S9B). Collectively, the data indicate that FER is a key molecular contributor to melanoma cell dissemination and formation of metastatic foci at distant sites, independent of its effects on tumor growth and endpoint tumor size. 

## 3. Discussion

To investigate the role of FER kinase in melanoma, we developed two distinct FER loss-of-function models based on human melanoma cell lines that have the capacity to metastasize in vivo. These cells are invaluable tools to better understand the molecular basis of metastatic behavior in this tumor type, especially given the low survival rate of patients with metastatic disease [35]. 

A key finding of our studies is that FER is required for efficient dissemination of melanoma cells to distant sites, through mechanisms independent of tumor ability to invade locally. The pro-metastatic effects of FER are likely to result from its coordinate modulation of several, distinct pathways. First, FER is necessary for normal melanoma cell proliferation and transit through S-phase. This is consistent with the reported role of FER in breast carcinoma cells, in which FER downregulation causes increases in the G0/G1 cell fractions, with concomitant decreases in S-phase cells [20]. Our functional proteomics screen revealed that cyclin D3 is downregulated in FER knockdown cells. Cyclin D3 is overexpressed in melanoma cells relative to normal melanocytes, and it is necessary for G1 to S-phase progression [36]. The contribution of cyclin D3 to FER regulation of melanoma cell proliferation remains an important area for future investigation. 

Second, FER-deficient melanoma cells seeded on laminin 332 show deficits in motility. We observed significant decreases in accumulated distance migrated in both KO and KD cell line models. However, the effect of FER silencing in 131/4-5B1 cells on mean speed and Euclidean distance migrated was negligible, if any, possibly due to the remaining low levels of FER protein in these cells. Melanoma cell interactions with ECM proteins present in the skin, such as collagen IV and laminin 332, are important for cell migration and invasion. We have observed that both normal primary cultured melanocytes and parental 131/4-5B1 melanoma cells exhibit greater motility on laminin 332 compared to collagen I ([26] and Figure S2). Similarly, A375 and other melanoma lines show enhanced haptotactic migration in response to laminin 332, relative to collagen and other ECM substrates, a response likely mediated through α3β1 integrins [37]. Laminin 332-induced A375 melanoma cell migration was reportedly abrogated upon blockade of integrin α3β1, and interactions between laminins and integrins appear to be important for metastatic foci formation in melanomas [37]. FER has been implicated in the synthesis of laminin-binding glycans, which normally results in increased cell attachment, at the expense of cell migration on this substrate [38]. In breast carcinoma cells, FER silencing induces focal adhesion formation, increased cell adhesion and decreased migration through upregulation of integrins α6 and β1 [20]. Whether similar mechanisms are implicated in the positive modulation of melanoma cell migration by FER remains to be investigated. 

At the molecular level, we identified L1-CAM and β-catenin signaling as FER downstream targets that potentially contribute to metastasis of melanoma tumors. L1-CAM protein levels were similarly reduced in both FER iKD and FER KO melanoma cells, indicating that the extent of FER downregulation in response to silencing by shRNA treatment is sufficient to induce biological alterations that are very similar to those observed upon *FER* gene inactivation. In the high L1-CAM expressor 131/4-5B1 line, we were able to detect decreases in both steady-state mRNA levels and apparent protein half-life upon FER silencing, suggesting that FER modulates L1-CAM pre- and post-translationally. Significantly, L1-CAM has been implicated in melanoma progression [31], and is a key positive modulator of metastasis in both wild-type and BRAF^V600E^ melanomas [39].

FER activates β-catenin-associated transcription directly through Tyr142 phosphorylation [40] and indirectly through various mechanisms that lead to decreased β-catenin degradation [41]. Survival of metastatic melanoma cells, but not of primary melanocytes, is β-catenin dependent [42]. Further, Wnt/β-catenin signaling enhances human melanoma cell migration and invasion in vivo, both in NRAS-driven mouse models of melanoma and in the neural crest of chick embryos [43,44]. FER may contribute to melanoma metastasis through phosphorylation and consequent inhibition of the low-density lipoprotein receptor-related protein 6 (LRP6). LRP6 activation increases β-catenin degradation and is associated with reduced 5-year survival in melanoma patients [45,46]. We found that FER depletion in melanoma cells also disrupted the ability of the degradation-resistant β-catenin S33Y mutant to activate TCF/LEF activity. This effect was not associated with detectable perturbation of nuclear import of the β-catenin mutant. Thus, FER may also regulate the ability of nuclear β-catenin to associate with TCF/LEF and/or to form transcriptionally-active complexes with these factors. Collectively, our studies suggest that FER likely promotes melanoma metastasis through the sum of its effects on various distinct cellular pathways.

The mechanisms involved in FER regulation of tumorigenesis likely differ depending on tumor type. In support of this notion, our protein array studies revealed no detectable changes in phosphorylation of the Met receptor tyrosine kinase, which has been implicated as a key direct FER target essential for metastasis of ovarian carcinoma [22]. Thus, although FER appears to be a critical pro-metastatic factor in various neoplasms, targeted approaches to interfere with these properties would need to take into account the identity of downstream pathways activated in individual tumor types.

In conclusion, the present study has demonstrated that interfering with FER functions effectively reduces the growth and metastatic potential of melanoma, coordinately interfering with several pathways known to be pro-metastatic in this tumor. The development of agents that target FER phosphotransferase or scaffolding activity may provide the basis for therapeutic interventions to combat metastatic neoplasm dissemination. 

## 4. Materials and Methods 

### 4.1. Plasmids and Lentivirus

The following plasmids were obtained from Addgene (Cambridge, MA, USA): pUltra-Chili (48687) and pUltra-Chili-Luc (48688, gifts from Malcolm Moore), pSpCas9(BB)-2A-GFP (48138, gift from Feng Zhang [47]), pcDNA3-S33Y β-catenin (19286, gift from Eric Fearon [48]), and phL1A-pcDNA3 (12307, gift from Vance Lemmon [49]). M50 Super 8x TOPFlash (12456) and M51 Super 8x FOPFlash (12457, gifts from Randall Moon [50]) contain, respectively, eight copies of consensus or mutant TCF/LEF DNA binding sites upstream from sequences encoding firefly luciferase [50]. The *Renilla* luciferase-encoding plasmid pRL-CMV (E2261) was purchased from Promega (Madison, WI, USA). The small hairpin RNA (shRNA) transfer vectors containing control or FER-targeting shRNA sequences (FER[978-998]) were generated as described [20,51]. Lentiviruses encoding GFP and dox-inducible control or FER-targeting shRNAs were packaged in COS-7 cells, as described [52]. To generate lentivirus encoding firefly luciferase and tdTomato fluorescent protein, COS-7 were transfected with pUltra-Chili-Luc and lentivirus packaging plasmids, as described [52].

### 4.2. Cell Culture, Lentiviral Transduction and FER Gene Editing 

Melanoma tumor biopsies were obtained after informed consent from patients and processed according to ethical protocol HSREB#103381, which was approved by the Ethics Committee of the University of Western Ontario and the London Health Sciences Centre. Biopsies were dissected by a pathologist immediately after surgery. Primary melanoma cells were isolated and cultured, and total cell lysates were prepared as described [53].

A375-MA2 (CRL-3223) and COS-7 (CRL-1651) cells were purchased from the American Type Culture Collection and maintained in DMEM supplemented with 8% fetal bovine serum (FBS) at 37 °C in a humidified atmosphere containing 5% CO_2_. 131/4-5B1 cells [23] were a generous gift from Dr. Robert Kerbel (Sunnybrook Health Sciences Centre, Toronto, ON, Canada). The BRAF^V600E^ mutation is present in both A375-MA2 and 131/4-5B1 cells [23,24]. 131/4-5B1 cells were profiled by short tandem repeat analysis and maintained in RPMI 1640 medium with L-glutamine and 5% FBS. Polyclonal 131/4-5B1 control and FER inducible knockdown (iKD) cell lines were generated by transduction of the parent 131/4-5B1 line with lentivirus encoding GFP and dox-inducible control or FER-targeting shRNA [51]. GFP-positive cells were isolated 48 h after transduction by fluorescence-activated cell sorting (FACS). To silence the *FER* mRNA, cells were cultured in the presence of 2 μg/ml dox for 120 h. The dox-containing growth medium was replaced every 48 h to maintain shRNA expression. To generate polyclonal control and FER iKD luciferase-expressing (Luc+) cell lines, 131/4-5B1 control and FER iKD cells were transduced with lentivirus encoding firefly luciferase and tdTomato fluorescent protein. GFP- and tdTomato-positive cells were isolated 48 h after transduction by FACS. 

CRISPR/Cas9-mediated gene editing was used to generate FER knockout (KO) A375-MA2 cells. Single guide (sg) RNAs targeting exon 1 or exon 3 of the *FER* gene were designed with the CRISPR Design Tool (http://tools.genome-engineering.org). The following oligonucleotides (targeting sequences are underlined) were annealed and cloned into the *BbsI* site of pSpCas9(BB)-2A-GFP to generate plasmids containing sgRNAs targeting *FER* exon 1 or exon 3:

- FER exon 1 coding 5′-CACCGAGACTGGGAATTACGGTTAC-3′
- FER exon 1 non-coding 5′-AAACGTAACCGTAATTCCCAGTCTC-3′
- FER exon 3 coding 5′-CACCGGCTTTGTCGTATCGTTCCT-3′
- FER exon 3 non-coding 5′- AAACAGGAACGATACGACAAAGCC-3′

A375-MA2 cells were transfected with pSpCas9(BB)-2A-GFP only (Cas9 control) or pSpCas9(BB)-2A-GFP containing *FER* exon 1- or exon 3-targeting sequences, using Lipofectamine 2000 (11668030, Thermo Fisher Scientific, Burlington, ON, Canada). GFP-positive cells were isolated 48 h after transfection by FACS and seeded at single-cell density in 96-well plates. Single cell-derived clones were expanded, genomic DNA was isolated and regions containing the target sites were amplified by polymerase chain reaction (PCR) using the following primer sets:

- FER exon 1 forward 5′-GGCTAAGACCTCTGGAGTAATGTT-3′
- FER exon 1 reverse 5′-TCTCCATGAGATTTTCATACTCTGTA-3′
- FER exon 3 forward 5′-AGTATCCTACATGCCTCCTTGGCAA-3′
- FER exon 3 reverse 5′-CAACTCTCTGGAGGCAGATATTCAC-3′

Amplicons were resolved on 3.5% native polyacrylamide gels to identify the presence of indels, as described [54]. Clonal cells that were positive for indels were further screened by immunoblot analysis to confirm the absence of FER protein. The FER KO 1 and 2 monoclonal lines, containing indels in FER exon 1 and 3, respectively, were selected for all further studies. To minimize phenotypic drift, cells in culture were replenished after 10–12 passages from frozen stocks. All cell lines used in these studies were verified to be mycoplasma-free using the MycoAlert Plus mycoplasma detection kit (LT07-701, Lonza, Basel, Switzerland).

### 4.3. Immunoblot Analysis

Cell lysates were prepared and analyzed as described [55]. Tumor tissue lysates were prepared by homogenizing in 2x lysis buffer (10 mM Tris, 150 mM NaCl, 1 mM EDTA, 1 mM EGTA, 1% Triton X-100, 0.5% NP-40, 1 mM PMSF, 5 mM Na3VO4, 1 μg/ml aprotinin, 1 μg/ml pepstatin, 1 μg/ml leupeptin) and incubated on ice for 30 min. The lysates were then centrifuged at 10,000× *g* for 10 min at 4 °C. The primary antibodies and dilutions used were: mouse anti-FER (4268S, Cell Signaling Technology, Pickering, ON, Canada; 1:500), mouse anti-CD171 (L1-CAM) (MA5-14140, Thermo Fisher Scientific; 1:200), mouse anti-γ-tubulin (T6557, Sigma, St. Louis, MO, USA; 1: 50,000). Proteins were detected using horseradish peroxidase-conjugated goat anti-mouse IgG (115-038-003, Jackson ImmunoResearch, West Grove, PA, USA; 1:500) and Clarity Western ECL Substrate detection reagent (170-5060, Bio-Rad, Hercules, CA, USA). Immunoblot images were acquired using a VersaDoc Imaging System (Bio-Rad) and Quantity One software (version 4.6.9, Bio-Rad). Densitometric analyzes were conducted using Image Lab software (version 6.0.0, Bio-Rad). 

### 4.4. Immunofluorescence Microscopy

Cell cultures were fixed in freshly diluted 4% paraformaldehyde (PFA) and processed for immunofluorescence microscopy as described [56]. The primary antibodies used were: mouse anti-5-bromo-2’-deoxyuridine (BrdU, G3G4, Developmental Studies Hybridoma Bank (DSHB), Iowa City, IA, USA, deposited to the DSHB by Kaufman, S.J.; 1:200), rabbit anti-Ki67 (15580, Abcam, Cambridge, MA, USA; 1:500), mouse anti-β-catenin (610154, BD Biosciences, San Jose, CA, USA), mouse anti-Pmel17 (sc-377325, Santa Cruz, Dallas, TX, USA). Secondary antibodies purchased from Thermo Fisher Scientific were: AlexaFluor 555-conjugated goat anti-mouse IgG (A-21422; 1:500) and AlexaFluor 555-conjugated goat anti-rabbit IgG (A-21428; 1:500). Nuclear DNA was visualized with Hoechst 33342 (H1399, Thermo Fisher Scientific, Burlington, ON, Canada; 100 ng/ml). Fluorescence photomicrographs were obtained with a Leica DMIRBE fluorescence microscope equipped with an Orca-ER digital camera (Hamamatsu Photonics, Hamamatsu City, Japan), using Volocity 6.1.1 software (Improvision-PerkinElmer, Waltham, MA, USA). 

To detect GFP and collagen type III in tissue specimens, antigen retrieval was conducted by boiling for 20 min in 10 mM citrate buffer, pH 6.0, followed by a 30-min cooling period. The chicken anti-GFP antibody (13970, 1:500) was from Abcam. The anti-collagen type III mouse monoclonal antibody (3B2; deposited to the DHSB by R. Mayne; 1:6) was obtained from the DHSB. Secondary antibodies: AlexaFluor 647-conjugated goat anti-chicken IgG (A-21449) and AlexaFluor 555-conjugated goat anti-mouse IgG (A-21422, Thermo Fisher Scientific) were both used at a 1:500 dilution. 

### 4.5. Cell Motility Assays

Cells were plated on 35-mm µ-Dish culture plates (81156, ibidi, USA Inc., Madison, WI, USA) that were coated with laminin 332 matrix [57], at a density of 1 × 10^4^ cells/cm^2^. Images of cells cultured at 37 °C in a humidified atmosphere containing 5% CO_2_ were acquired over a 16-h period, using an EVOS Cell Imaging System equipped with an EVOS onstage incubator (Thermo Fisher Scientific), and capturing a phase-contrast image every 10 min. Image sequences were imported into Fiji software [58] and the Manual Tracking Plug-in was used to track every cell within each frame. Migration tracks were then analysed using the Chemotaxis and Migration Tool software (ibidi). The migration path of each cell was visually outlined, and the accumulated (total) distance, the euclidean distance (straight linear distance from the initial to the final cell position point) and the average speed of each cell were calculated.

### 4.6. Cell Adhesion Assays

Wells of 96-well tissue culture plates were coated with laminin 332 matrix for 30 min at 37 °C. Asynchronously growing melanoma cells were trypsinized and re-plated at 1 × 10^4^ cells/well. After 30, 60, 90 or 300 min of incubation at 37 °C, unattached cells were removed by rinsing the wells with PBS, followed by addition of culture medium. Then, cells were fixed in 4% PFA for 20 min at 22 °C, rinsed once with PBS and stained with 0.2% crystal violet for 2 min. Cells were washed twice with PBS and the crystal violet was extracted in 100% methanol for 10 min. OD_562_ was determined in triplicate wells and the percentage of adhered cells was calculated. 

### 4.7. Reverse Phase Protein Array (RPPA) Analysis

Control and FER iKD 131/4-5B1 cells were cultured in medium with or without 2 μg/mL dox for 120 h. Duplicate cell samples were collected, and flash frozen in liquid N_3_. Cell lysates were analysed at the RPPA core facility of the MD Anderson Cancer Center (Houston, TX, USA).

### 4.8. Gene Expression Analysis

Total RNA was isolated using RNeasy mini kits (74104; Qiagen, Hilden, Germany), and cDNA was synthesized from 1 µg of total RNA, using iScript cDNA synthesis kits (1708890, Bio-Rad). Quantitative, real-time polymerase chain reactions (qPCR) were conducted on a CFX96 Real-Time PCR Detection system (Bio-Rad). cDNA samples corresponding to 20 ng of RNA were amplified using TaqMan Fast Advanced Master Mix (4444556, Thermo Fisher Scientific). *L1-CAM* transcript levels were analysed by using a Taqman assay (ID: Hs01109748_m1; 4331182). *HPRT1* (ID: Hs02800695_m1; 4448484) and *HMBS* (ID: Hs00609296_g1; 4448484) mRNAs were used as references. The data were analysed with CFX Manager software (version 3.0, Bio-Rad), using the ΔΔC_q_ method.

### 4.9. Measurement of TCF/LEF Activity

Cells were seeded in triplicate wells on 96-well plates, at a density of 5000 cells/well. Twenty-four hours after plating, the cells were transfected using Lipofectamine 3000 (L3000-001, Thermo Fisher Scientific). For TCF/LEF reporter activity assays, cells were transfected with either 45 ng M50 Super 8× TOPFlash or M51 Super 8× FOPFlash plasmids, together with 45 ng pcDNA3 or pcDNA3-S33Y β-catenin, as well as 10 ng of pRL-CMV. Firefly luciferase reporter activity was measured 48 h post-transfection using the Dual-Glo Luciferase Assay System (E2920, Promega), and was normalized to *Renilla* luciferase.

### 4.10. Ex Ovo CAM Assays

Fertilized chicken eggs (McKinley Hatchery, St. Mary’s, ON, Canada) were incubated in a rotary incubator (Berry Hill, St. Thomas, ON, Canada) at 37 °C and 70% humidity for 3 days, after which the eggs were cracked, and the embryos (at day 4 of gestation) and CAM were placed into sterile weigh boats that were covered with a plastic lid allowing air circulation. The weigh boats were placed in a plastic rack that contained autoclaved water and were maintained in a stationary incubator set at 37 °C and 70% humidity. On day 7 post incubation, 1.5 × 10^6^ control or FER iKD 131/4-5B1 cells that had been pre-treated with dox for 5 days were suspended in 30 μL of Cultrex Basement Membrane Matrix (3632-001-02, Trevigen, Gaithersburg, MD, USA) were grafted onto a branching vascular point of the CAM. The grafted embryos were incubated in the stationary incubator for 7 days, at which time tumors and surrounding CAM were excised and fixed overnight in 4% PFA to prepare for further processing and analysis.

### 4.11. In Vivo Xenograft Tumorigenesis Studies

All experiments using mice were approved by the University of Western Ontario Animal Care Committee (Protocol No. 2017-061), in accordance with regulations and guidelines from the Canadian Council on Animal Care. Female, 8–10-week-old NOD.Cg-*Prkdc^scid^ Il2rg^tm1WjI^*/SzJ immunodeficient (NSG) female mice were obtained from the Humanized Mouse and Xenotransplantation Facility at the Robarts Research Institute, University of Western Ontario, London, Canada. Approximately 1.25 × 10^5^ firefly luciferase-expressing 131/4 5B1 control or FER iKD cells were injected subcutaneously on the flank of the animals. Tumor growth was measured using a digital caliper on a weekly basis. Mice were fed dox-containing chow (S3888; Bio-Serv, Flemington, NJ, USA) for the duration of the experiment. For bioluminescence imaging (BLI), mice received IP injections of D-luciferin (122799, PerkinElmer, Waltham, MA, USA; 150 mg/kg dissolved in PBS). BLI was conducted weekly, using an IVIS Lumina XRMS In Vivo Imaging System (CLS136340, PerkinElmer) operated with Living Image software (128110, PerkinElmer). Average radiance (photons/second/mm^2^/steradian) of tumors and distant metastases was measured with region-of-interest (ROI) analysis using LivingImage Software. Mice were euthanized as soon as tumor volume (V = 4/3 × π × (L/2) × (W/2)^2) attained 1000 mm^3^ or when BLI revealed the presence of metastases, after which time the primary tumor and organs were harvested for further analysis.

### 4.12. Immunohistochemistry (IHC)

Tissues were fixed in 4% PFA and processed at the Molecular Pathology Core Facility, Robarts Research Institute, University of Western Ontario, London, Canada. Paraffin-embedded sections (6 µm) were dewaxed, rehydrated and stained with Mayer’s Hematoxylin (MHS16) and Eosin Y (HT110116), purchased from Millipore Sigma (Oakville, ON, Canada). Melanin in tissue sections was visualized using Fontana-Masson (ab150669, Abcam), as described [26]. Coverslips were mounted onto slides using Permount (SP15100, Fisher Scientific, Hampton, NH, USA). 

### 4.13. Statistical Analysis

Statistical analyses were conducted using GraphPad Prism software (version 7.03). In vivo BLI tumor growth data were analyzed with two-way repeated measures ANOVA and post hoc Sidak’s test. BLI distant metastases data were analyzed with Welch’s t test. For survival analysis, the Kaplan-Meier method was used. All other data were analyzed with one-way ANOVA and post-hoc Tukey’s test. All data are presented as the mean ± SEM. Significance was set at *p* < 0.05. 

## 5. Conclusions

In this study, we found that FER kinase regulates melanoma cell migration and metastatic dissemination through multiple mechanisms, including activation of β-catenin/TCF-LEF and L1-CAM signaling. 

## Supplementary Materials

The following are available online at https://www.mdpi.com/2072-6694/11/3/419/s1, Figure S1: FER kinase mutations and expression in clinical melanoma samples, Figure S2: FER regulates melanoma cell motility, Figure S3: Effect of FER downregulation on melanoma cell-matrix adhesion, Figure S4: Reverse phase protein array (RPPA) analysis of 131/4-5B1 melanoma cells, Figure S5: Effect of FER downregulation of L1-CAM protein stability, Figure S6: Expression of S33Y β-catenin in A375-MA2 melanoma cells, Figure S7: Illustration of the chicken chorioallantoic membrane (CAM) used to measure 131/5-5B1 cell invasion, Figure S8: Generation of firefly luciferase-expressing control and FER inducible knockdown (iKD) 131/4-5B1 melanoma cells, Figure S9: Effect of FER downregulation on melanoma primary tumor growth.
