# Supplementary material for: Targeting FER Kinase Inhibits Melanoma Growth and Metastasis
Source: Cancers (Basel). 2019 Mar 24;11(3):419. doi: 10.3390/cancers11030419 (PMC6468679; doi:10.3390/cancers11030419)
Supplement: Supplementary file 1 [file cancers-11-00419-s001.pdf]

Supplementary Figure S1

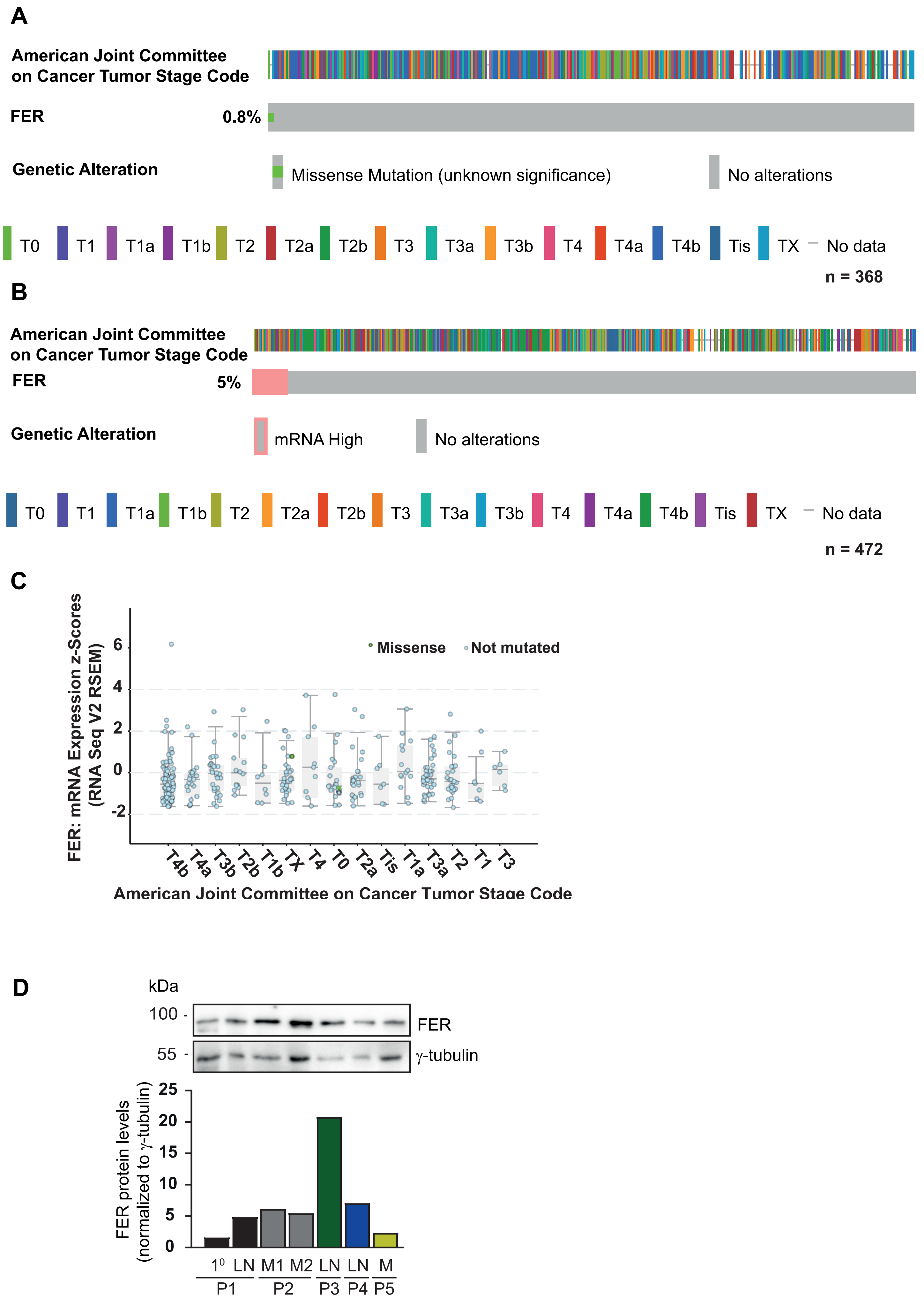

Supplementary Figure S1. FER kinase mutations and expression in clinical melanoma samples. (A) Analysis of FER mutations in melanoma tumors from The Cancer Genome Atlas (TCGA) database. **(B and C)** Analysis of FER mRNA expression in melanoma tumors from the TCGA database showing that FER expression is not significantly different across melanoma stages (denoted according to the Cancer Tumor Stage Code). **(D)** FER protein levels in primary cells derived from biopsies of melanoma tumors of five patients (P1-P5) were detected by immunoblot. γ-tubulin was used as a loading control. Primary tumor – 1^0; lymph node metastasis – LN; distant metastasis – M.

Supplementary Figure S2

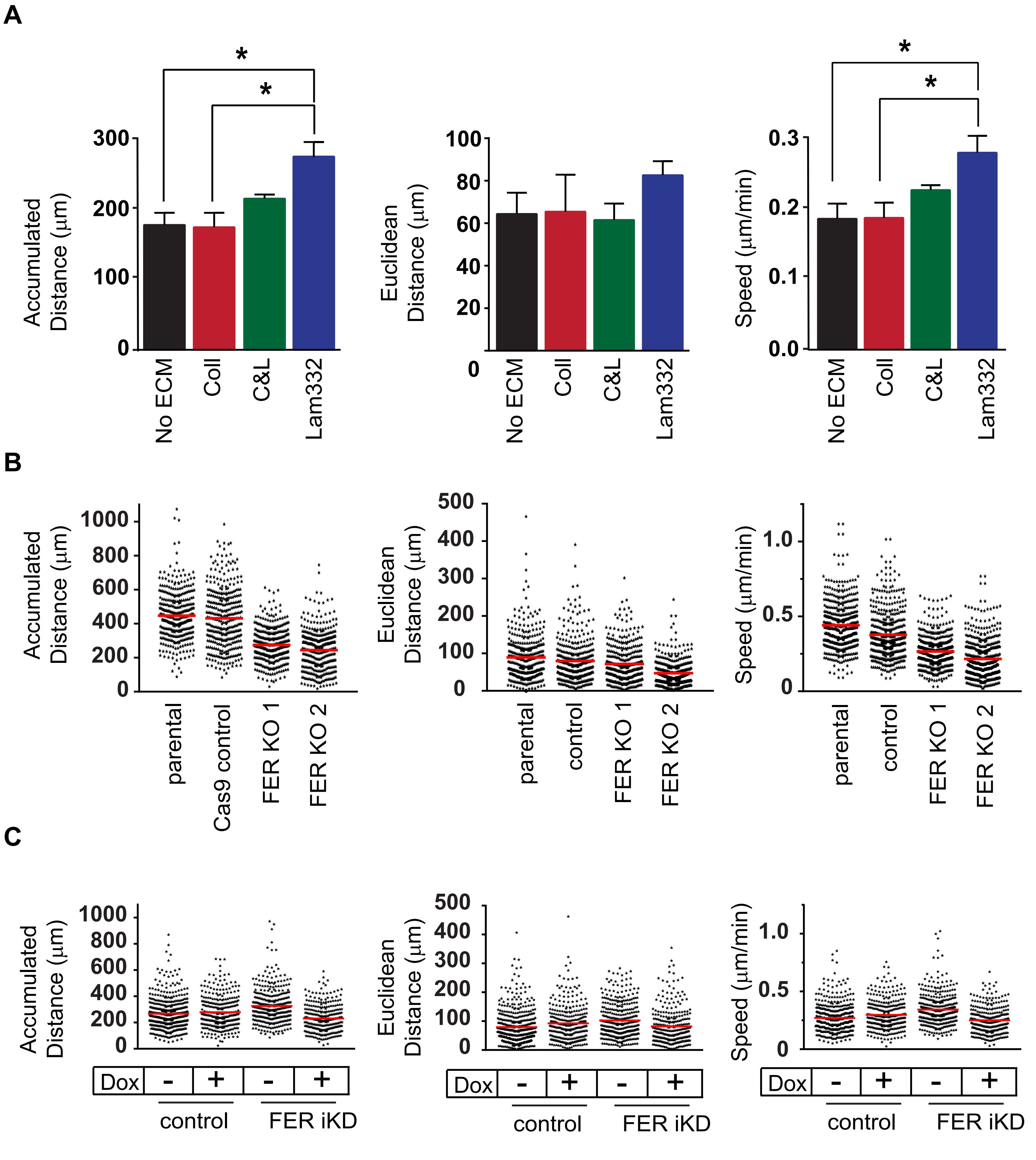

Supplementary Figure S2. FER regulates melanoma cell motility. (A) Untreated 131/4-5B1 cells were plated on μ-Dishes without any exogenous extracellular matrix (No ECM) or coated with collagen I (Coll), laminin 332 (Lam332) or both (C&L). Cells were imaged for 16 h using time-lapse videomicroscopy and analysed using Image J (NIH) and the Chemotaxis and Migration Tool Software (ibidi). Two hundred cells were tracked in total for each cell group. Histograms represent mean ± SEM (N=3) accumulated (total) distance, Euclidean distance (straight linear distance from the initial to final migratory point) and speed. * represents $P < 0.05$ (ANOVA). **(B)** A375-MA2 parental, Cas9 control and FER KO cells were cultured in medium containing 0.5% FBS for 96 h or **(C)** Control and FER iKD 131/4-5B1 cells were cultured in medium with or without 2 μg/ml of dox for 120 h. Then, cells were cultured on μ-Dishes coated with laminin 332. Cells were imaged for 16 h using time-lapse videomicroscopy and analysed using Image J (NIH) and the Chemotaxis and Migration Tool Software (ibidi). Dot plots show the distribution of accumulated distance, Euclidean distance and speed values for individual cells. Red lines represent the mean for each population.

Supplementary Figure S3

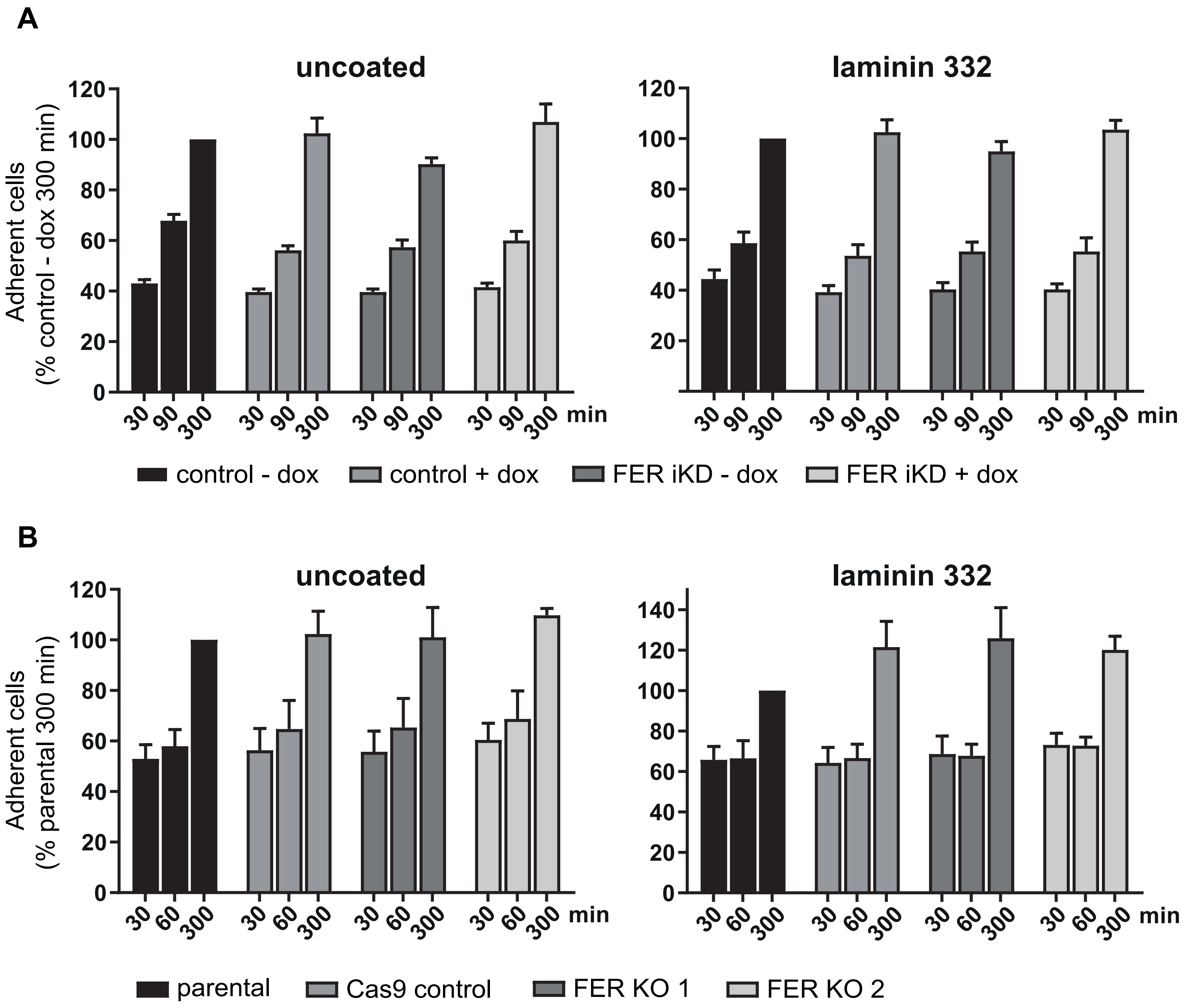

Supplementary Figure S3. Effect of FER downregulation on melanoma cell-matrix adhesion. **(A)** 131/4-5B1 control and FER inducible knockdown (iKD) cells were cultured in medium with or without 2 µg/ml of dox for 120 h or **(B)** A375-MA2 parental, Cas9 control and FER KO cells were cultured for 24 h. Then, cells were plated on 96-well plates in triplicate (uncoated or coated with laminin 332) for the indicated times and the percentage of attached cells was determined. Values represent the relative proportion of attached cells ± SEM in three independent experiments.

Supplementary Figure S4

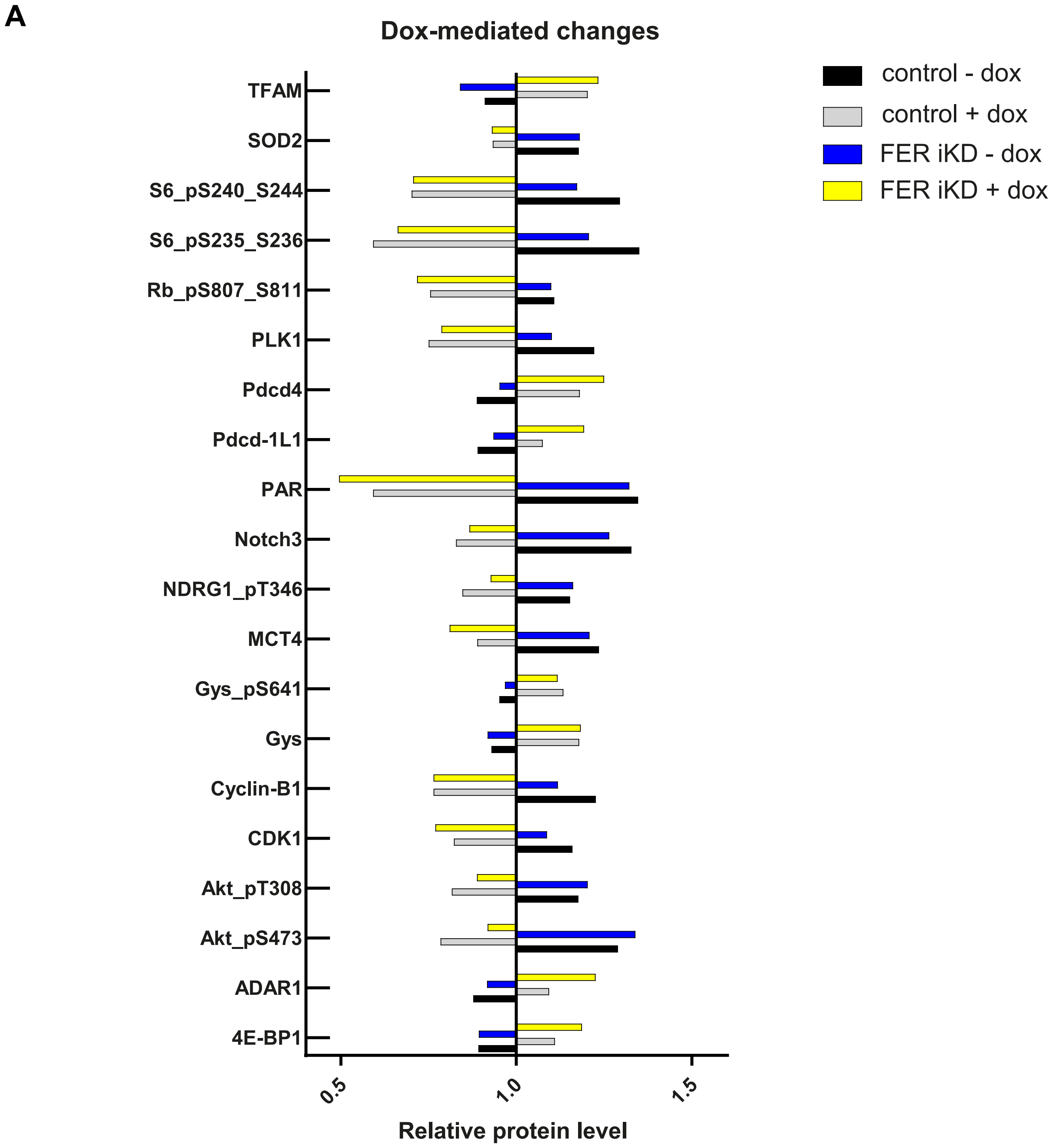

B

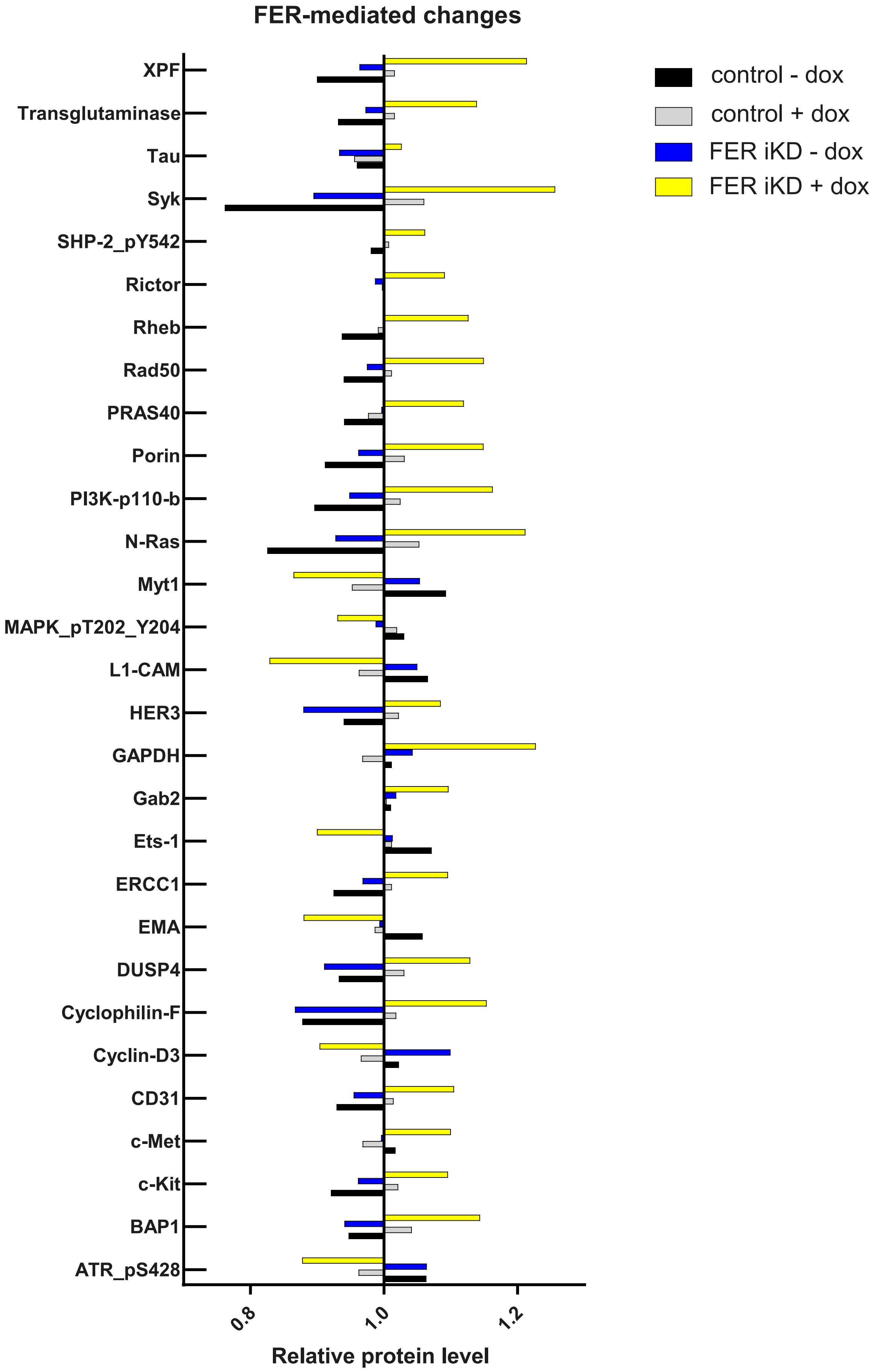
FER-mediated changes
XPF
Transglutaminase
Tau
Syk
SHP-2_pY542
Rictor
Rheb
Rad50
PRAS40
Porin
PI3K-p110-b
N-Ras
Myt1
MAPK_pT202_Y204
L1-CAM
HER3
GAPDH
Gab2
Ets-1
ERCC1
EMA
DUSP4
Cyclophilin-F
Cyclin-D3
CD31
c-Met
c-Kit
BAP1
ATR_pS428
0.8
1.0
1.2
Relative protein level
control - dox
control + dox
FER iKD - dox
FER iKD + dox

Supplementary Figure S4. Reverse phase protein array (RPPA) analysis of 131/4-5B1 melanoma cells. 131/4-5B1 control or FER iKD melanoma cells were cultured for 120 h in the absence (-) or presence (+) of 2 µg/ml doxycycline (dox) and the levels of 306 proteins in lysates from duplicate samples were determined by using RPPA analysis. **(A)** Normalized levels of proteins affected by dox treatment. Uncommon abbreviations: 4E-BP1 - eukaryotic translation initiation factor 4E-binding protein 1; ADAR1 - RNA-specific adenosine deaminase 1; Gys - glycogen synthase; MCT4 - monocarboxylate transporter 4; NDRG1 - N-Myc downstream regulated 1; PAR - poly-ADP ribose polymer; Pdcd-1L1 - programmed cell death 1 ligand-1; Pdcd4 - programmed cell death 4; PLK1 - Polo-like kinase 1; S6 - S6 ribosomal protein; SOD2 - superoxide dismutase 2; TFAM - mitochondrial transcription factor A. **(B)** Normalized levels of proteins affected by FER depletion. Uncommon abbreviations: ATR - *ataxia telangiectasia*-mutated and Rad3-related kinase; BAP1 - BRCA1-associated protein 1; DUSP4 - dual specificity protein phosphatase 4; EMA - epithelial membrane antigen; ERCC1 - excision repair cross-complementation group 1; Gab2 - GRB2-associated-binding protein 2; GAPDH - glyceraldehyde 3-phosphate dehydrogenase; HER3 - human epidermal growth factor receptor 3; L1-CAM - L1-cell adhesion molecule; Myt1 - membrane-associated tyrosine- and threonine-specific cdc2-inhibitory kinase; PRAS40 - proline-rich Akt substrate of 40 kDa; Rheb - Ras homolog enriched in brain; Rictor - rapamycin-insensitive companion of mTOR; SHP-2 - Src homology phosphatase 2; Syk - spleen tyrosine kinase; XPF - *xeroderma pigmentosum* group F-complementing protein.

Supplementary Figure S5

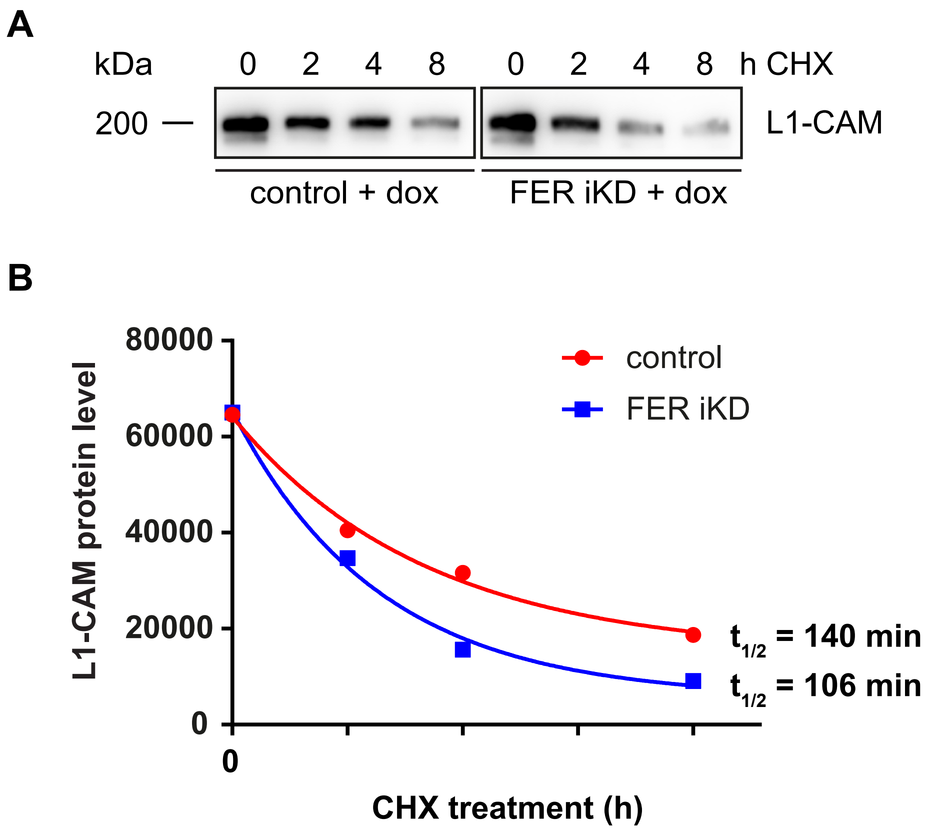

Supplementary Figure S5. Effect of FER downregulation of L1-CAM protein stability. (A) 131/4-5B1 control or FER iKD melanoma cells were cultured for 120 h in the presence of 2 µg/ml doxycycline (dox). Then, cells were treated with 100 µg/ml cycloheximide (CHX) at timed intervals and L1-CAM levels in cell lysates were determined by immunoblot. **(B)** L1-CAM levels were quantified for each time point and the protein half-life of L1-CAM was calculated.

Supplementary Figure S6

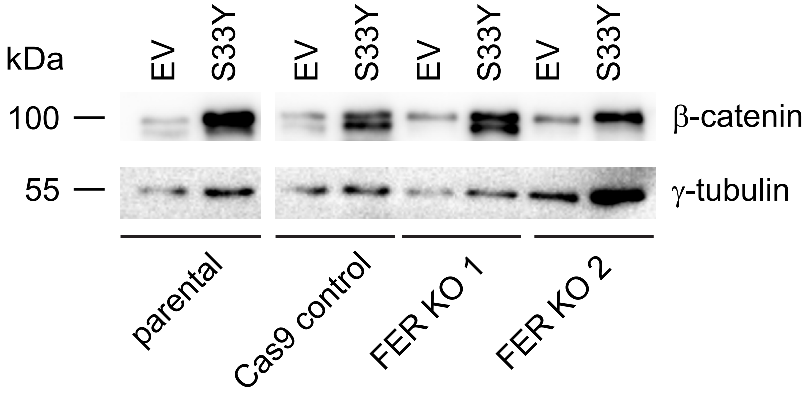

Supplementary Figure S6. Expression of S33Y β-catenin in A375-MA2 melanoma cells. A375-MA2 parental, Cas9 control and FER KO cells were transfected with pcDNA3.1 (empty vector; EV) or vector expressing a stable β-catenin S33Y mutant. β-catenin in cell lysates was detected by immunoblot. γ-tubulin was used as a loading control.

Supplementary Figure S7

A

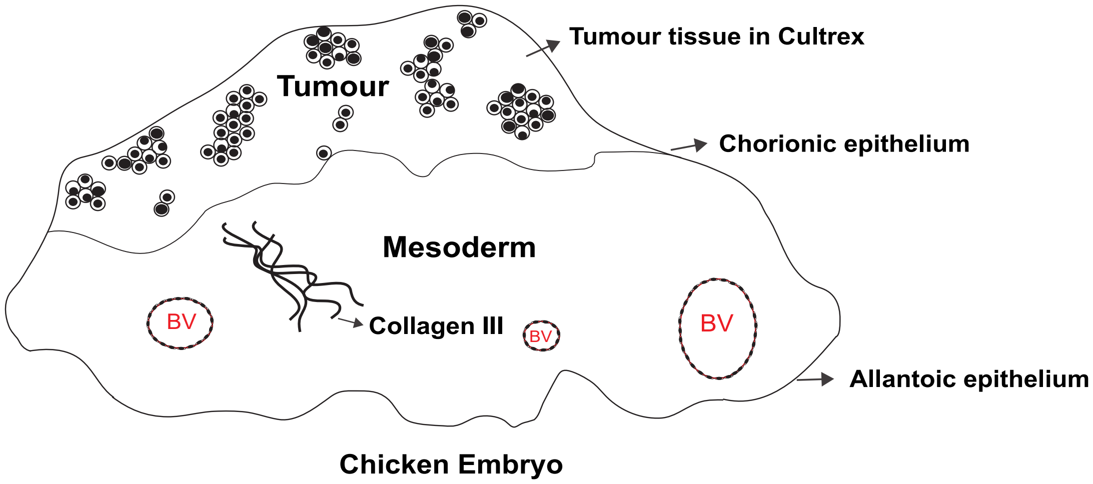

B

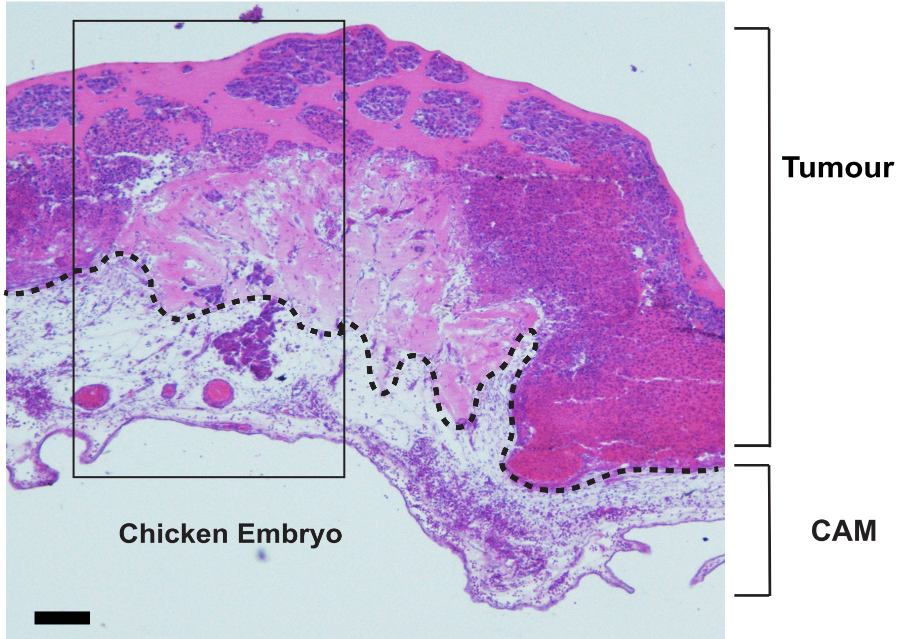

C

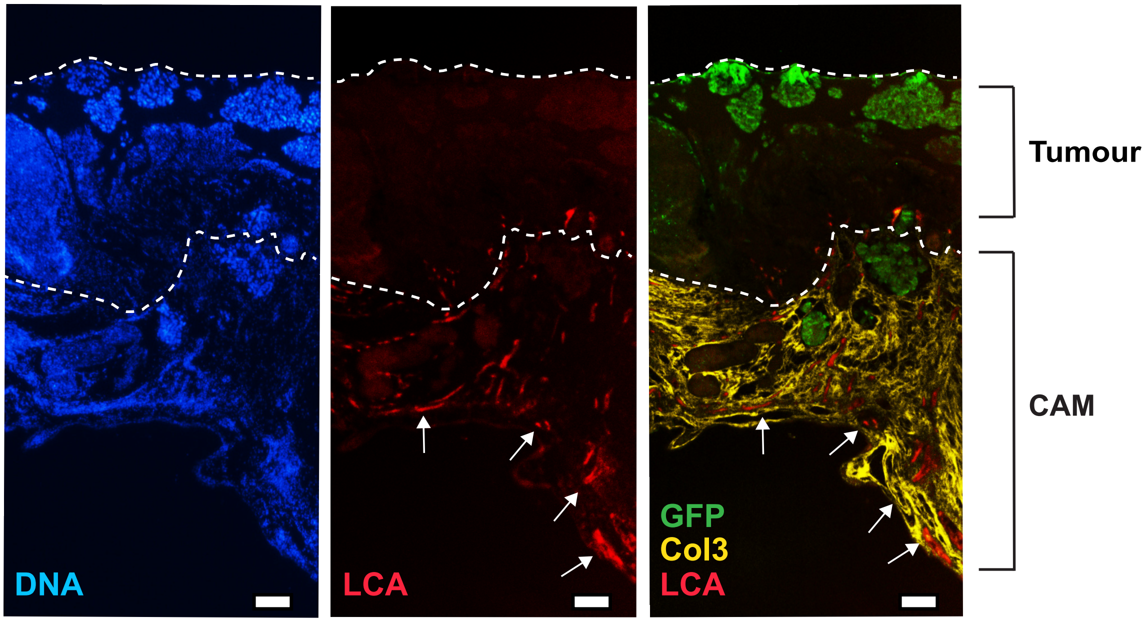

D

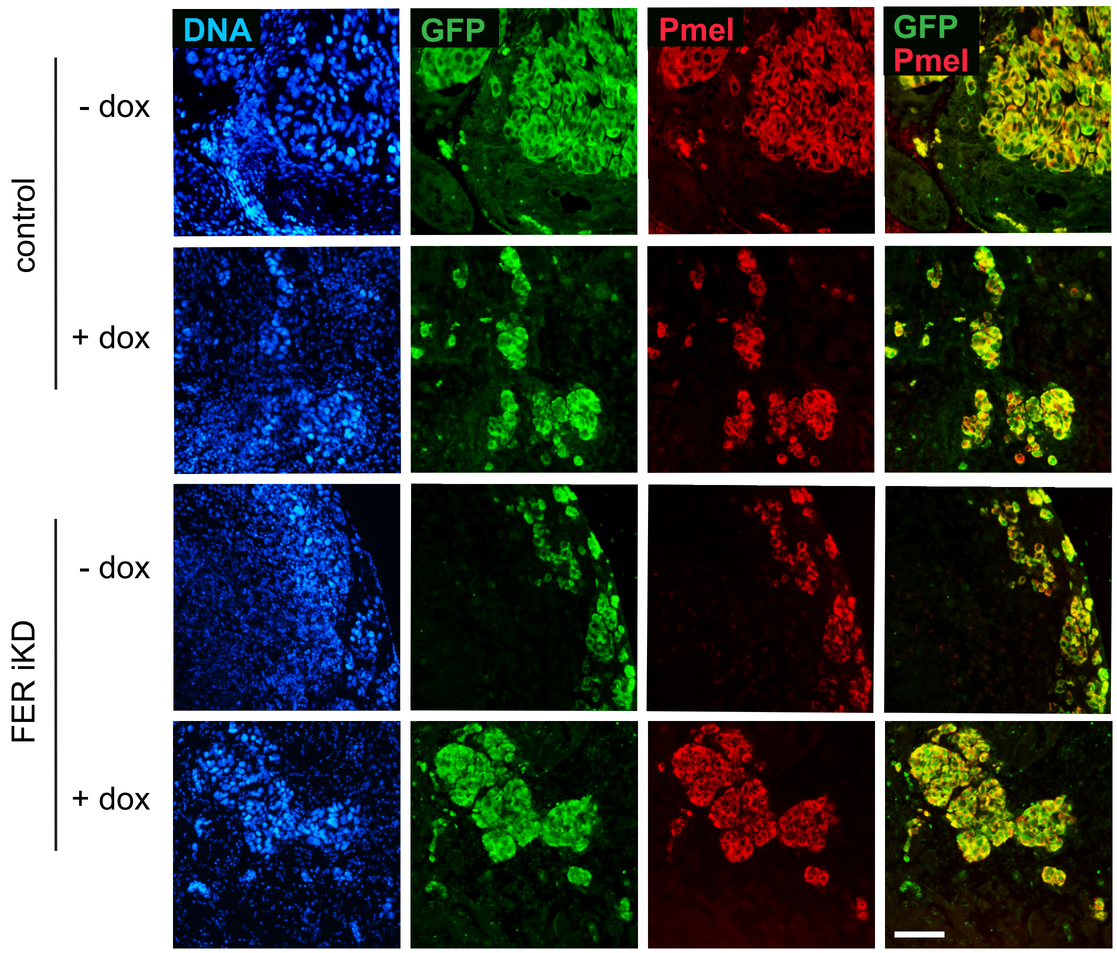

Supplementary Figure S7. Illustration of the chicken chorioallantoic membrane (CAM) used to measure 131/5-5B1 cell invasion. 131/4-5B1 control and FER inducible knockdown (iKD) cells were cultured in medium with or without 2 μg/ml of dox for 5 days. Cells suspended in Cultrex were grafted on the CAMs of embryonic day 11 (E11) embryos and incubated for an additional 7 days to allow for tumour growth. On E18, rhodamine labelled *lens culinaris agglutinin* (LCA) was injected into a blood vessel of the CAM, tumours were excised and processed for further analysis. **(A)** Schematic of tumour growth on the CAM. BV = blood vessel. **(B)** Representative hematoxylin and eosin (H&E) stained tumour tissue section showing tumour growth and invasion into CAM mesoderm. Black Bar = 100 μm. The area outlined by the black rectangle is represented in **(C)** as fluorescence images demonstrating 131/4-5B1 cell detection using GFP immunoreactivity and detection of the CAM using a chicken-specific collagen III antibody. Shown in red, are the endothelial cells labelled with rhodamine-LCA. White bar = 100 μm. **(D)** GFP and PMEL immunoreactivity in control and FER iKD tumour tissue to confirm the melanocytic lineage of GFP-positive 131/4-5B1 cells. White bar = 64 μm.

Supplementary Figure S8

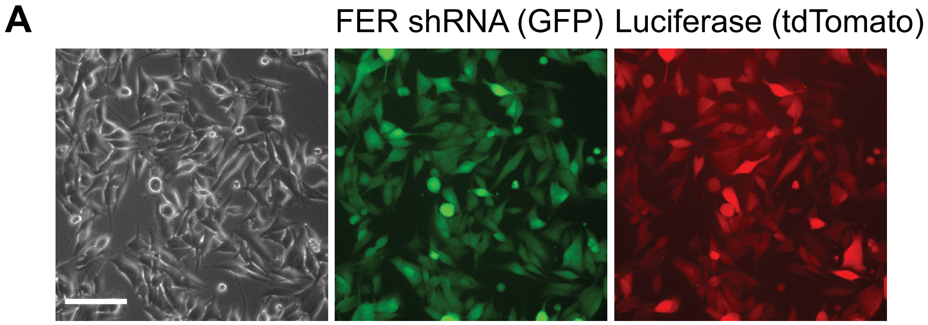

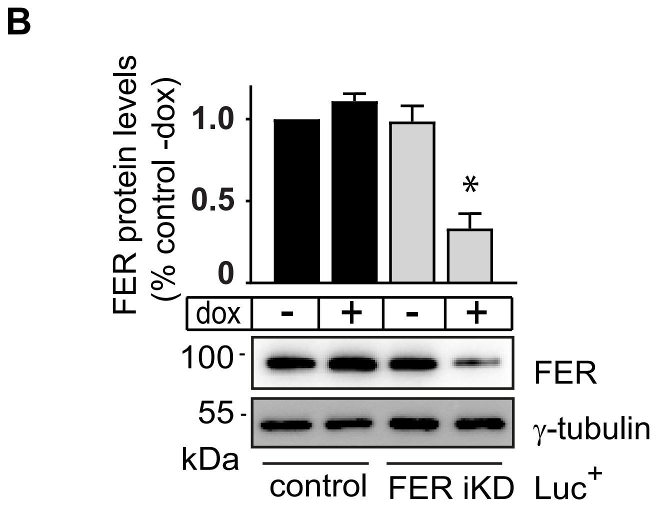

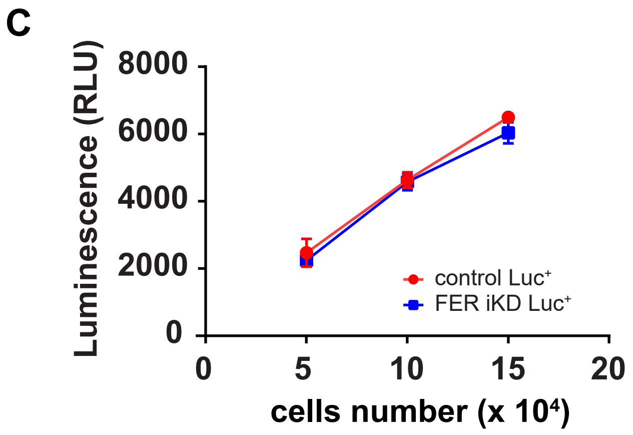

Supplementary Figure S8. Generation of firefly luciferase-expressing control and FER inducible knockdown (iKD) 131/4-5B1 melanoma cells. (A) 131/4-5B1 control and FER iKD cells were transduced with lentivirus encoding firefly luciferase and tdTomato fluorescent protein. Cells were FACS sorted to generate control and FER iKD luciferase-expressing (Luc+) cell lines. Immunofluorescence analysis showing that all cells express both GFP and tdTomato, indicating stable integration of the shRNA and firefly luciferase vectors, respectively. **(B)** Control and FER iKD_Luc+ cells were cultured for 120 h in the absence (-) or presence (+) of 2 µg/ml doxycycline (dox) and FER kinase was detected by immunoblot. γ-tubulin was used as a loading control. Histograms represent mean FER protein levels ± SEM (N=3) normalized to γ-tubulin and expressed relative to control - dox cells. **(C)** Control and FER iKD_Luc+ cells were seeded on 24-well plates in duplicate wells and luciferase activity was measured after 4 h by using the BrightGlo kit (E2610, Promega). The graph shows mean luciferase levels at the indicated cell densities ± SD (N=3).

Supplementary Figure S9

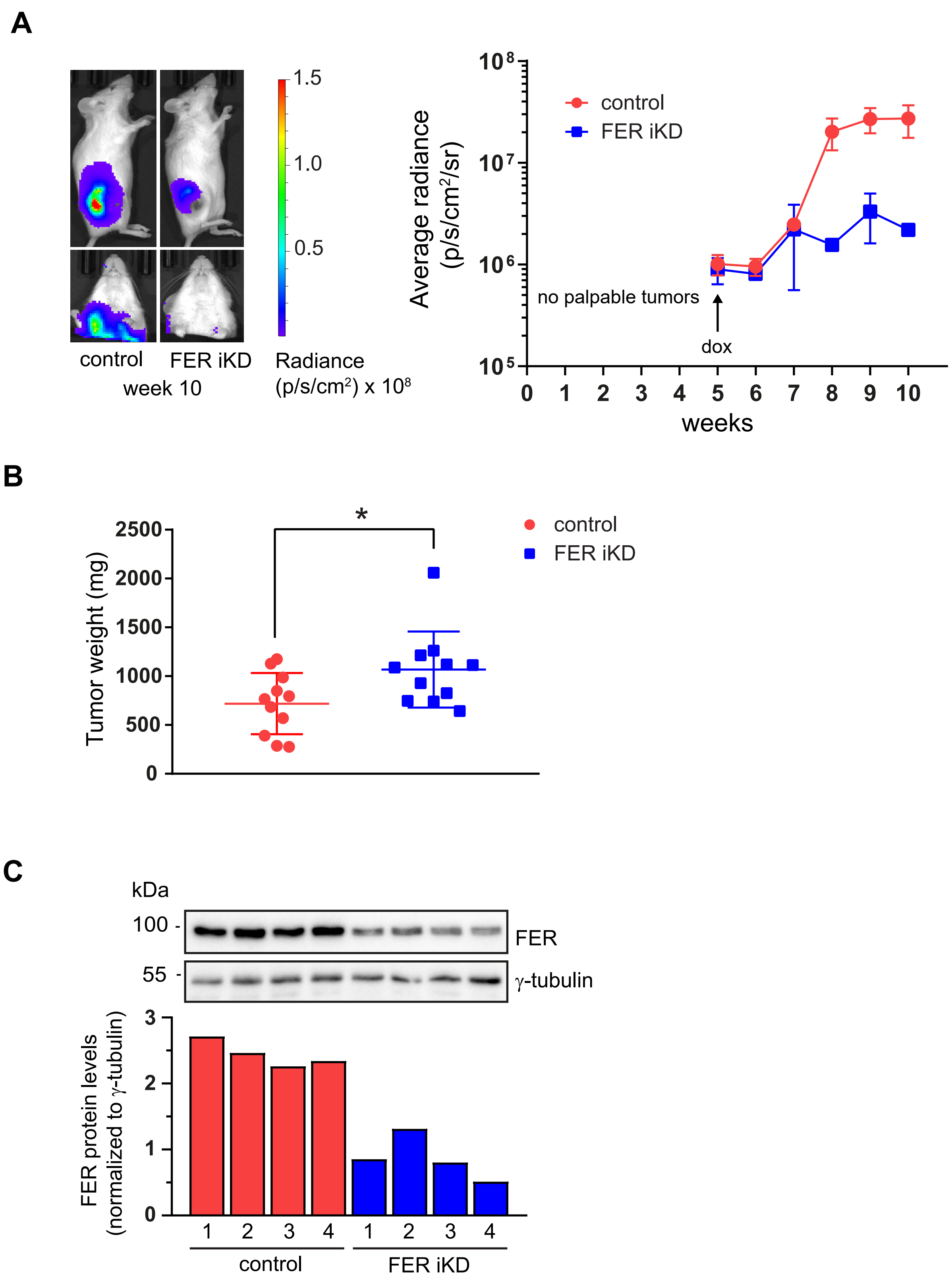

Supplementary Figure S9. Effect of FER downregulation on melanoma primary tumor growth. (A) Bioluminescence imaging (BLI) of melanoma cells *in vivo*. Luciferase-expressing control and FER inducible (iKD) melanoma cells were orthotopically transplanted in NSG immunodeficient mice. Once the tumors were palpable, the mice were fed a dox-containing diet to induce shRNA expression. Cells were detected in live animals using BLI. The graph shows the average radiance in primary tumors measured weekly after the start of dox administration (arrow). Lung metastases were detected only in control tumor-bearing mice (n = 2). **(B)** Luciferase-expressing control and FER iKD melanoma cells were orthotopically transplanted in NSG immunodeficient mice, which were fed a dox-containing diet to induce shRNA expression. Tumor weights were measured at endpoint (weeks 8-10 and 11-13 for control and FER iKD tumor-bearing mice, respectively. n = 11). * represents $P < 0.05$ (unpaired t-test). **(C)** FER protein levels in tumor samples from NSG mice transplanted with control and FER iKD melanoma cells were detected by immunoblot. γ-tubulin was used as a loading control.
